# Novel isothiacalothrixin B analogues exhibit cytotoxic activity on human colon cancer cells *in vitro* by inducing irreversible DNA damage

**DOI:** 10.1371/journal.pone.0202903

**Published:** 2018-09-06

**Authors:** Nachiappan Dhatchana Moorthy, Bose Muthu Ramalingam, Saleem Iqbal, Arasambattu K Mohanakrishnan, Krishnasamy Gunasekaran, Elangovan Vellaichamy

**Affiliations:** 1 Department of Biochemistry, University of Madras, Guindy Campus, Chennai, India; 2 Department of Biotechnology, Orchid Pharma Limited, Chennai, India; 3 Department of Organic Chemistry, University of Madras, Guindy Campus, Chennai, India; 4 Center for Advanced studies in Crystallography & Biophysics, University of Madras, Chennai, India; VIT University, INDIA

## Abstract

Preliminary cytotoxic analysis of sulphur containing isosteric analogues of calothrixin B identified the useful anti-tumour activity of thia/isothiacalothrixin B which necessitated it’s biological evaluation in colon and lung cancer cell lines. The isothia analogues induced cytotoxicity of HCT116 in a time-dependent manner and inhibited the clonogenic survival of HCT116 and NCI-H460 cells in a dose-dependent manner comparable to the standard anti-cancer drug camptothecin. Herein employing flow cytometry, we demonstrate that isothiacalothrixin B analogues inhibited proliferation of colon cancer cells by the arrest of cells in S and G2/M phases over a period of 48 hours at a concentration of 5 μM. Our results also suggest that the cytotoxicity of thia analogues of calothrixin B is partially mediated by induction of cellular DNA strand breaks. The UV-Vis spectroscopic studies with CT-DNA revealed groove binding for calothrixin B and its thia analogues wherein subsequent *in silico* molecular modelling studies indicated preferential binding to the AT-rich regions of minor groove of DNA. Furthermore, thiacalothrixin B caused transcriptional activation of p21^waf1/cip1^ promoter and upregulation of its protein levels independent of p53. The induction of DNA damage response pathway leads to apoptosis in isothiacalothrixin B but not in thiacalothrixin B treated cells. The isothia analogues **SCAB 4** induced DNA strand breaks and cell cycle arrest even after treatment for a short period (i.e., 4 hours) and the cell cycle effects were irreversible. For the first time, this study provides detailed cellular effects on the potential use of isothiacalothrixin B analogues as cytotoxic agents.

## Introduction

The naturally occurring quinocarbazole alkaloid namely, calothrixin B was initially identified to possess nanomolar growth inhibitory action against HeLa cells [1], and recently, it was reported to exert its anti-proliferative activity in a panel of cancer cell lines differing in p53 status [2]. Although calothrixin B is less potent than its N-oxide bearing calothrixin A, an attempt to introduce fluorine substituent to the E-ring of calothrixin B resulted in 3-fluoro calothrixin B having superior cytotoxic profile comparable to calothrixin A [2]. One useful approach of modulating the anticancer property involves the replacement of aromatic heterocyclic framework with the sulphur-containing heterocyclic unit which results in a change of regiochemical features such as size, shape and electron donating ability to assist proper fit of the ligand within the drug target [3]. The presence of sulfur atom in many experimental and clinically approved drugs have been widely reported in the literature where the low-lying C-S σ* orbitals in sulphur heterocyclics induce a positive electrostatic potential to favour non-bonding interactions with the biological targets [3, 4]. Earlier studies with isothiaellipticine, the sulphur isostere of the pyridocarbazole alkaloid ellipticine is capable of restoring p53 function in a manner analogous to ellipticine, the parent compound [5]. Also, the replacement of carbazole nitrogen (NH) with sulphur atom resulted in thia calothrixin B [**SCAB 1**] having a cytotoxic property inferior to calothrixin B, however upon its isomerisation yielded isothiacalothrixin B [**SCAB 4**] having superior anti-cancer potency in cancer cell lines [6]. The synthesis of isosteric analogues of calothrixn B is beneficial both in search of more active yet specific cytotoxic compounds and helps in explaining the mode of action of these compounds on cancer cell lines. In this report, we evaluated the cytotoxic potential of thia analogues of calothrixin B [**SCAB 1–12**] in HCT116 (colon cancer cells) and explored the possible mechanism of cell killing by these analogues. The results from our studies suggest that isothiacalothrixin analogues caused apoptotic cell death in HCT116 cells through induction of irreversible DNA damage.

## Materials and methods

### Reagents and antibodies

A detailed procedure on the preparation of calothrixin B and its sulphur analogues [**SCAB 1–12**], their characterization data (IR, NMR, MS and HRMS spectral analysis) were discussed in our earlier reports [2, 6]. The > 95% purity of the compounds (calothrixin B, **SCAB 1–12**) were determined by high-resolution mass spectral (HRMS) data. In this paper, we discuss the results from the biochemical studies of calothrixin B and its thia analogues (**SCAB 1–12)**. Sulphorhodamine-B (SRB), Crystal violet, Trypan blue, Propidium Iodide (PI), RNase, Dithiothreitol, Acridine Orange and Ethidium Bromide were purchased from Sigma (St. Louis, MO, USA). BCA protein assay kit, ECL plus chemiluminescence kit were purchased from Thermo Scientific Pierce. pGL4.2[*luc2/puro*] Vector, Reporter 5x lysis buffer and Luciferase assay reagents were purchased from Promega, Madison, WI, USA. Protease inhibitor cocktail (Cat. No.539134) and phosphatase inhibitor cocktail (Cat. No.524627) were purchased from Calbiochem, Merck-Millipore, CA, USA. The nitrocellulose membrane was obtained from Bio-rad. Specific antibodies against p53, p21^waf1/cip1^ and corresponding secondary antibodies were purchased from Cell Signaling Technology (Beverly, MA, USA). Antibody to β-actin was purchased from Sigma (St. Louis, MO, USA).

### Cell lines and cell culture

Human cancer cell lines HCT116 (colon), NCI-H460 (Lung), U251 (Glioma), MCF7, MDA-MB231 (Breast), HeLa and SiHa (Cervical) were purchased from American Type Culture Collection, ATCC. Cells were cultured in Dulbecco’s modified Eagle’s medium (DMEM, Invitrogen) supplemented with Heat inactivated 10% (v/v) fetal bovine serum (FBS, Invitrogen) and antibiotic solution (100 μg/ml streptomycin and 100 IU/ml penicillin), in a humidified atmosphere of 5% CO_2_ at 37 °C. The cells were subcultured and maintained in culture flasks following the instructions from ATCC.

### Sulphorhodamine-B cytotoxicity assay

The cell viability was measured by SRB assay following the NCI protocol [7, 8]. In brief, the cells were seeded into 96 well cell culture plates with seeding densities ranging from 1500–3000 cells /well. The thiacalothrixin B stock solution was prepared in DMSO at a concentration of 1 mM and subsequently diluted to working concentration of 4 μM in culture media. After over-night adherence, the cells were treated with different drug concentrations ranging from 4 μM to 0.2 nM for 48 hours. At the end of treatment period, cells were fixed with 10% trichloroacetic acid for 1 h at 4°C and washed with 1% acetic acid. The fixed cells were stained with 0.54% SRB for 15 min, washed three times and left to dry at room temperature. The plates were developed by solubilising the bound dye with a Tris-Base solution, and the absorbance was measured at 530 nm using a microplate reader. The half-Maximal growth inhibition (GI_50_) values which are the time zero corrected, reduction in cell number by 50% in comparison with that of untreated control was obtained, and standard deviation from at least two independent experiments was computed using Microsoft Excel.

### Clonogenic assay

The clonogenic assay was performed following established procedure [9] to assess the long-term effect of the reported analogues on cell viability. In brief, the colon (HCT116) and lung (NCI-H460) cancer cells were obtained as a single cell suspension and seeded at concentrations of 100 cells/well of a six-well plate (in triplicate). After over-night adherence, the cells were treated with different concentrations of calothrixin B and its thia analogues for 48 hours and then washed with Dulbecco’s Phosphate buffered saline. The cells were cultured in fresh medium without drugs for a period of 14 days until visible colonies formed which are then stained with crystal violet and photographed.

### Cell cycle analysis

The distribution of cells in different phases of cell cycle was determined by the phase distribution of DNA content using propidium iodide (PI) staining. For nocodazole experiment, HCT116 were treated with calothrixin B/thiacalothrixin B alone (5 μM) for 20 hours or pre-treatment of cells with calothrixins for 4 hours followed by 0.4μM nocodazole for additional 17 hours. In the case of DRC experiment, the cells were treated with different concentration (0.5, 1 and 2 μM) of thiacalothrixins and the adherent cells were collected omitting the floating cells at different time points (24 and 48 hours after treatment) by trypsinisation, washed twice with phosphate-buffered saline (PBS) and fixed with 70% ethanol. Before staining, cells were washed with PBS and stained with PI (20 μg/ml) in PBS containing Triton X-100 (0.1% v/v) and RNase A (0.2 mg/ml) for 1 h at room temperature. Cell cycle distribution was analyzed using a Beckman-Coulter flow cytometer.

### DNA cleavage studies

The ability of thiacalothrixins to cleave pBSK plasmid DNA was determined in the presence of Dithiothreitol (DTT) and ferric chloride by agarose gel electrophoresis [2]. In brief, the plasmid DNA (250 ng) was incubated at pH 8.0 in 20 mM HEPES buffered solution containing varying concentrations of calothrixins, 200 μM DTT and 200 μM FeCl_3_. The cleavage reactions were carried out at 37°C for 1-hour following which the addition of loading dye stopped the reaction and electrophoresed in 1.5% Agarose gel containing 0.5 μg/mL ethidium bromide at 100V in Tris-acetate-EDTA (TAE) buffer. Finally, the gel was photographed under UV light. The DNA bands (linear and supercoiled forms) from representative experiments were quantified using ImageJ.

### Alkaline COMET assay

The alkaline COMET assay was performed by following the reported procedure [10] with slight modification. Briefly, the cells were collected by trypsinisation at the end of drug treatment period, and a cell suspension containing 10^4^ cells in 150 μl pre-warmed low melting point (LMP) agarose (0.5% PBS) was made. The above cell suspension was rapidly spread on doubly-frosted microscope slides (Rohem, India) pre-coated with normal melting (NMP) agarose (1%) and covered with a coverslip. Once the gel got solidified following incubation of the slide at 0°C, the coverslip was gently removed, and the third layer of 100 μl NMP agarose (0.5% PBS) was added. The slides were then incubated in lysis buffer (2.5 M NaCl, 0.1 M EDTA, 10 mM Tris—HCl pH 10, 10% DMSO and 1% Triton X-100 both freshly added) for 1 hour at room temperature. The slides were transferred to electrophoresis tank containing electrophoresis buffer (0.3 M NaOH and 1 mM EDTA, pH >13) and incubated for 40 minutes at room temperature. Electrophoresis was then carried out at room temperature in fresh electrophoresis buffer for 24 min (0.7 V/cm; 300 mA). The slides were then dried at 4°C, stained with ethidium bromide solution (20 μg/ml) and photographed under a UV-fluorescent microscope (magnification, x100; Nikon, Japan). The comets were quantitated using Comet Score software (Rex Hoover, TriTek Corpn.) and are expressed in terms of percentage of DNA migrated from the comet head to the tail region. [11]

### DNA binding studies

The CT-DNA solution was prepared, and its concentration was determined from the UV absorbance at 260 nm using molar extinction coefficient ε_260_ = 6600 M^−1^ cm^−1^ following the protocol of Ghosh et al. [12, 13]. The absorbance at 260 and 280 nm was recorded to determine the purity of DNA solution where the A_260_/A_280_ ratio > 1.85 depicting that the DNA was sufficiently free from protein [14]. Various concentrations of DNA were used to obtain a varied molar ratio of the DNA-compound adduct. Absorbance values were recorded on a double beam UV-2250 UV—VIS spectrophotometer (Shimadzu, Japan). Absorption titration experiments were conducted by keeping the concentration of calothrixin B or its thia analogues as constant (3× 10^−5^ mol L^-1^) while varying the CT-DNA concentration from 0 to 1 × 10^−4^ mol L^-1^. Absorbance values were recorded after each successive addition of DNA solution.

### DNA-drug molecular docking studies—Binding energy

The target DNA 1DSC, an octamer complexed with actinomycin D was obtained from protein data bank (PDB) [15]. The structure of 1DSC and the nucleotide base pairs (5’-D(*GP*AP*AP*GP*CP*TP*TP*C)-3’) was used for the analysis wherein the co-crystallized ligand was removed from the above structure. The receptor DNA (1DSC) and the selected calothrixins such as calothrixin B, **SCAB 1–12** were taken for *in silico* docking studies. The Chemsketch software was used to draw the structure of calothrixins [16]. Following which their energies were minimized for each compound using UCSF Chimera [17] for flexible conformations of the compounds during the docking. The flexible docking study was carried out using Autodock v4.0. Essential hydrogen atoms, Kollman united atom type charges and solvation parameters were added with the aid of Autodock tools [18]. This server integrates Lamarckian genetic algorithms. Each docking experiment was derived from ten different conformations. The relative stabilities were evaluated using free energy simulations and their binding affinities. The interaction analysis were calculated and visualised through the PyMol [19].

### DNA-drug molecular studies—Binding mode

#### Preparation of the ligands and DNA

The calothrixin B and its thia analogues were sketched using sketched module embedded in Schrodinger suite and were taken for minimization using Ligprep module of Schrodinger 09 (Ligpep 2.3, Schrödinger Suite 2009) [20] where probable tautomeric and ionization states at pH = 7 ± 1 followed by minimization with OPLS3 force field (Ligpep 2.3, Schrödinger Suite 2015) [20]. The different oligomeric DNA viz PDB ID’s: 453D [21] was performed using protein preparation wizard of Schrodinger 09 where missing hydrogen, the bond order was assigned followed by energy minimization. The resultant PDB co-ordinates were taken for docking studies.

#### Molecular modelling

Molecular docking of calothrixin B and its thia analogues were used as ligands against 453D and 1D98 using Glide XP 5.8 programme [22, 23]. Glide XP employs an anchor-and-grow sampling approach and a different functional form for Glide Score. The docked conformation corresponding to the lowest free energy (or highest score) provided by Glide program was selected as the most probable binding pose of top calothrixins B and its thia derivatives.

#### Visualisation of docking results

Once the docking was performed, best poses for hydrogen bonding, hydrophobic and π-π interactions were analysed using Chimera Visualisation tool [17], PyMol version 1.3 (The PyMOL Molecular Graphics System;) [19] and Glide (Schrödinger, LLC, New York, NY, USA) programs [22, 23].

### Western blot analysis

Protein expression was determined by Western blot. In brief, at specific time points after drug treatment, the total proteins from cells were extracted using modified RIPA buffer (10 mM Tris-Cl (pH 8.0), 140 mM NaCl, 1 mM EDTA, 1% Triton X-100, 0.1% SDS) containing protease and phosphatase inhibitor cocktail. The protein content in the lysates was determined using BCA assay kit. 20 μg of protein was loaded per lane. Following separation by SDS-PAGE, the proteins were transferred onto a nitrocellulose membrane and blocked by 5% non-fat milk for 1 hour at room temperature. The membrane was incubated overnight at 4 °C with primary antibodies and with the appropriate horseradish peroxidase (HRP)-conjugated secondary antibody (1 hour at room temperature). An ECL chemiluminescent plus substrate kit was used for detection following manufacturer protocol.

### Fluorescence microscopy assay using acridine orange and ethidium bromide staining

The cellular morphology after treatment with thiacalothrixin B analogues was studied using a double staining procedure [Ethidium Bromide (EtBr) and acridine orange (AO)] using a fluorescent microscope (Nikon TiE attached with NIS-AR Software) according to the standard procedure [24]. In brief, HCT116 and NCI-H460 cells (10–50 x 10^3^ cells/well) were grown on a 12 well plate and cultured with different concentrations of thiacalothrixin analogues for 48 hours. After drug treatment, cells were washed with PBS and then stained with acridine orange (20 μg/mL in PBS) and ethidium bromide (40 μg/mL in PBS) for 10 minutes at room temperature in the dark. After staining, the cells were washed twice with PBS and observed under a UV-fluorescent microscope (magnification, x100; Nikon, Japan).

### DNA fragmentation assay

The procedure reported by Herrmann et al., [25] was followed adopting slight modification for the isolation and analysis of apoptotic DNA fragments. In brief, HCT116 cells were treated with thiacalothrixin B analogues at a concentration of 5 μM for 48 hours, harvested using trypsin. The detached cells were collected by centrifugation and washed twice with ice-cold PBS buffer. The cells were then lysed using a buffer containing 1% NP-40 in 20 mM EDTA, 50 mM Tris-HCl, pH 7.5. The lysate is then centrifuged at 3000 rpm for 5 minutes to get clear supernatant to which 10 μl of 10% SDS and 10 μl of 5 mg/ml RNase A were added and incubated at 56°C for 90 minutes followed by digestion with proteinase K (final concentration 2.5 μg/μl) for at least 2 hours at 37 °C. DNA was isolated from the above mixture by the standard phenol-chloroform extraction method. The DNA pellets obtained after 70% ethanol wash step was air dried at room temperature for a few minutes and re-suspended in 50 μL of Tris-Cl pH 8.0 buffer. The prepared DNA samples were loaded onto a 1.5% gel electrophoresis, stained with ethidium bromide and the band obtained was visualised using the UV Gel Documentation system.

### Luciferase promoter analysis

HCT116 cells were transiently transfected with different expression constructs (p21P-*luc2* and p21PΔp53-*luc2*) using lipofectamine 3000 following the manufacturer’s protocol. After transfection, the cells were detached using mild trypsinisation procedure and seeded at a density of 20 X 10^4^ cells per well of a 96 well plate. Sixteen hours after transfection, the cells were exposed to different concentrations of thiacalothrixin B analogues for 48 hours. Firefly luciferase activities present in the cellular lysates were determined using luciferase assay reagent, and the light emission was quantitated using Spectramax microplate reader (Molecular Devices, San Diego, USA). Luciferase activities were normalised based on total protein concentrations. The p21^waf1/cip1^ promoter constructs used for the study were created in the following way. The promoter of human p21^waf1/cip1^ between positions -2300 and +8 contained in a plasmid pWWP-*luc* was a gift from B. Vogelstein (Johns Hopkins Univ., Baltimore, MD, USA) [26]. The *Hind*III promoter fragment (-2300/+8) was subcloned to pGL4.2 (*luc2/puro*) vector to produce p21P-*luc2*. The minimal promoter construct p21PΔp53-*luc2* was made by subcloning the *Kpn*1–*Hind*III fragment from positions −300 to +8 to pGL4.2 (*luc2/puro*) vector.

### Statistical analysis

Data were expressed as Mean ± S.D. from at least three independent experiments. Data were statistically analyzed using student t-test test in Graph-Pad Prism 5.0 software (Graph-Pad Software, Inc, San Diego, CA, USA) with values of *P < 0*.*05* being considered statistically significant.

## Results

### Isothiacalothrixins showed enhanced cytotoxic potential in human cancer cell line panel

**Fig 1:**
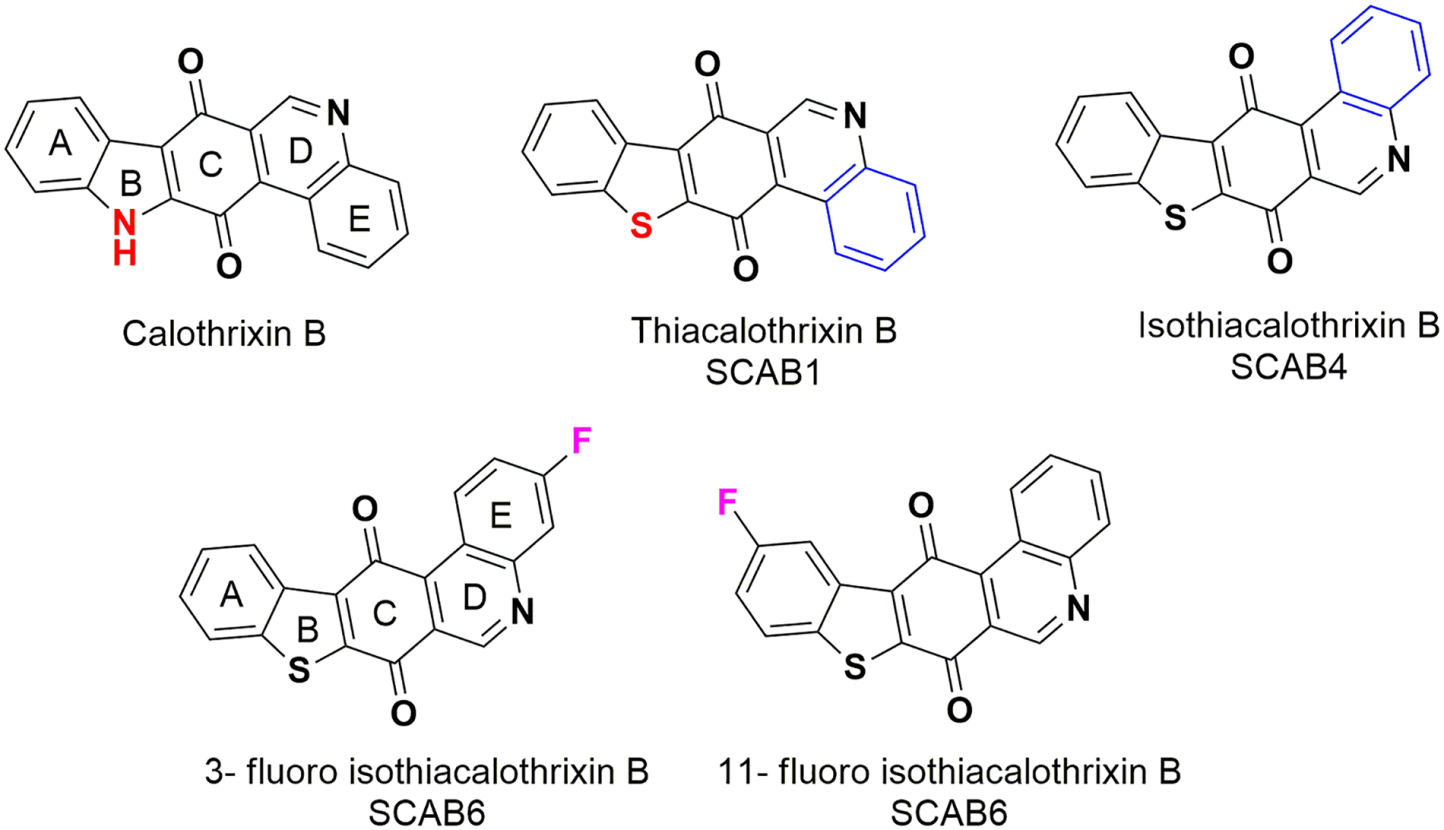
Chemical structures thia/isothiacalothrixin B analogues. The calothrixin B (**CAB 1**) is the naturally occurring anti-cancer alkaloid from the cyanobacteria *Calothrix*. The carbazole nitrogen group (-NH) was replaced with sulphur atom to result in thiacalothrixin B (**SCAB 1**) which upon isomerization results in isothiacalothrixin B (**SCAB 4**). The substitution with fluorine atom on the E-ring or A-ring of isothiacalothrixin B results in **SCAB 6** and **11**, respectively.

**Fig 2:**
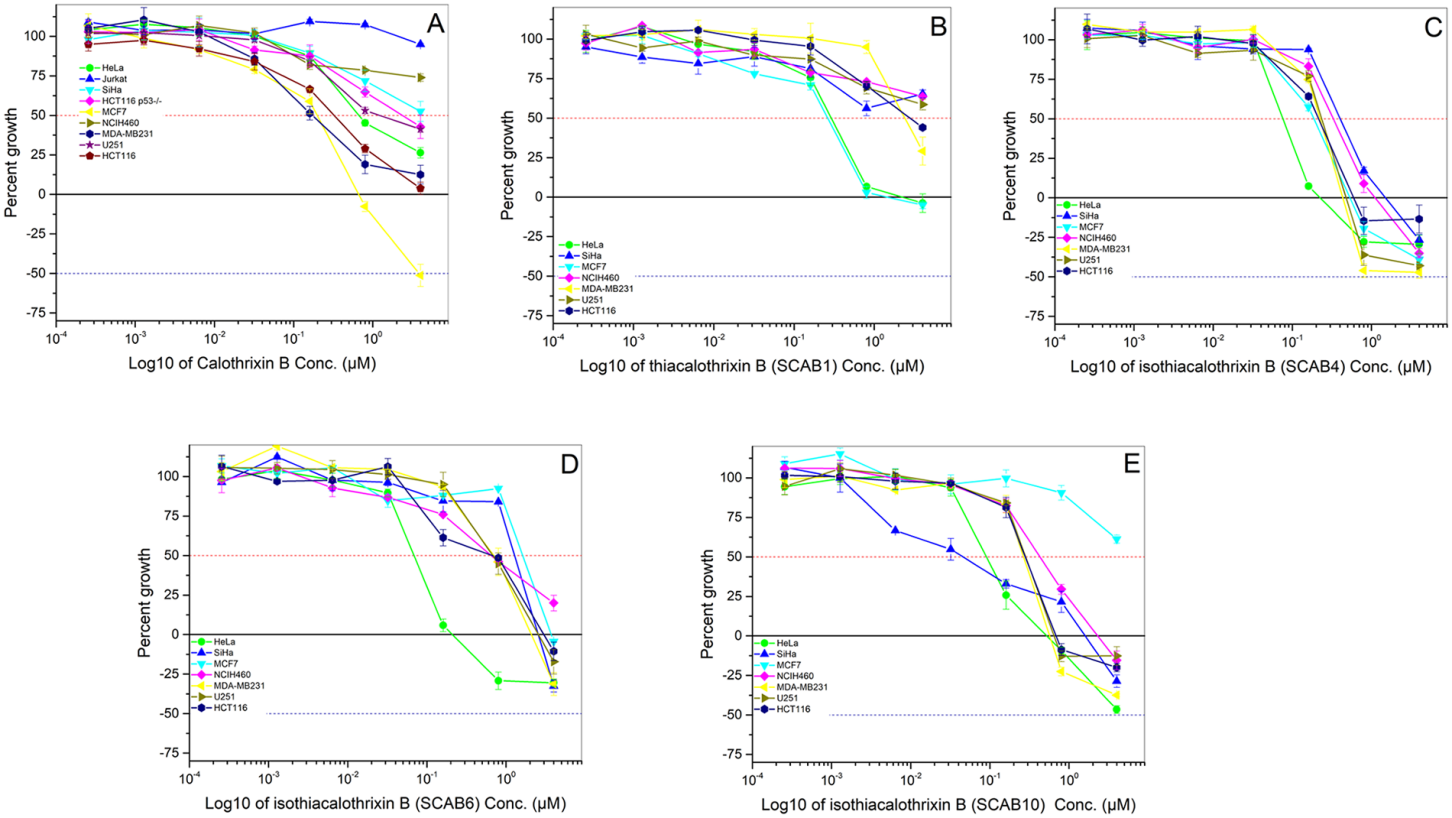
Isothiacalothrixins caused potent growth inhibition of cancer cell lines. Dose-response graphs of calothrixin B analogues assayed in a panel of nine cancer cell lines. Overlays of the percent growth of all nine cell lines following a 48 hours treatment with varying doses of calothrixin B (A), thiacalothrixin B (B), isothiacalothrixin B (C), 3-Fluoro isothiacalothrixin (D), 11-Fluoro isothiacalothrixin (E). HeLa (solid green circle), SiHa (solid blue triangle), MCF7 (solid cyan inverted triangle), NIC-H460 (solid magenta diamond), MDA-MB231 (solid yellow left-sided triangle), U251 (solid yellow right-sided triangle), HCT-116 (solid navy hexagon).

The cytotoxicity of thia analogues of calothrixin B (Fig 1 and S1 Table) were evaluated in a panel of tumour cell lines of different tissue origin in a serial 5-fold titration (4 μM to 2 nM) where the parent analogue calothrixin B and commonly used cytotoxic agent camptothecin was set for direct comparison. Cell death was assessed using Sulphorhodamine-B (SRB) assay. The GI_50_ value is the concentration of 50% of maximal inhibition of cell proliferation after 48 hours of drug treatment to cancer cell lines. The GI_50_ values of the promising analogues are summarised in Table 1, and the corresponding growth curves are represented in Fig 2. The replacement of indole nitrogen atom of naturally occurring calothrixin B with sulphur atom resulted in thiacalothrixin B (**SCAB 1**) having reduced cytotoxic profile. However, isomeric isothiacalothrixin B (**SCAB 4**) and its fluorinated analogues (**SCAB 6 & 10**) exhibited enhanced potency (almost >5 fold) in comparison to calothrixin B as well as thiacalothrixins B which was, comparable to the reference drug camptothecin. These results suggest that the profound antineoplastic activity of isothiacalothrixin B analogues are related to the isomeric modification of thiacalothrixin B (Fig 1). The cervical carcinoma cell line HeLa was found to be highly sensitive to thia analogues whereas no definitive trend towards sensitivity was displayed by other cancer cell type taken in the study.

**Table 1:** *In vitro* cytotoxicity data for calothrixin B and its thia analogues against seven human tumour cell lines.

Compound Name			Average GI_50_ ± S.D. (μM)^a^				
	HeLa	SiHa	HCT116	MDA-MB 231	U251	MCF7	NCI-H460
Calothrixin B (**CAB 1**)	0.47 ±0.09	3.50± 0.71	0.65±0.07	0.16±0.02	2.25±0.35	0.26±0.01	>4
Thiacalothrixin B (**SCAB 1**)	0.29 ±0.03	>4	1.10±0.00	>4	>4	>4	>4
isothiacalothrixin B (**SCAB 4**)	0.07±0.02	0.35 ± 0.07	0.23±0.03	0.22±0.06	0.25±0.05	0.23±0.04	0.28±0.09
3- Fluoro-isothiacalothrixin B (**SCAB 6**)	0.06 ±0.00	0.34 ±0.02	0.50±0.00	0.70±0.10	0.67±0.12	1.6±0.10	0.65±0.07
11- Fluoro-isothiacalothrixin B (**SCAB 1**0)	0.09 ±0.01	0.33 ±0.08	0.28±0.04	0.30±0.00	0.30±0.00	1.9±0.24	0.40±0.00
Camptothecin	0.19 ±0.03	0.55 ±0.00	0.05±0.01	0.4±0.01	0.01±0.01	0.06±0.00	0.006±0.003

^a^. Values represent mean from at least two independent experiment

### Isothiacalothrixins induces cytotoxicity in HCT116 cells in a time-dependent manner

Since the isothia analogues were potent in inhibiting cell proliferation at 48 hours post-treatment to HCT116 cells, the cytotoxic effect was examined beyond 48 hours utilising trypan blue exclusion assay. The cytotoxic effect of thia analogues of calothrixin B on HCT116 cells was studied at a dose of 5 μM for 24, 48, 72 and 96 hours post-treatment. Data obtained from three independent experiments indicated a time-dependent increase in cell death following treatment with isothiacalothrixin B analogues, but not in the parent thiacalothrixin B treated HCT116 cells (S1 Fig). Further, the percentage of live cell population reduced below 25% after 96 hours of treatment with isothiacalothrixin B analogues. Thus, there is a potent inhibition of HCT116 cell proliferation by isothia analogues which resulted in the maintenance of cytotoxicity over an extended period.

### Isothiacalothrixins inhibits colony formation in cancer cells

**Fig 3:**
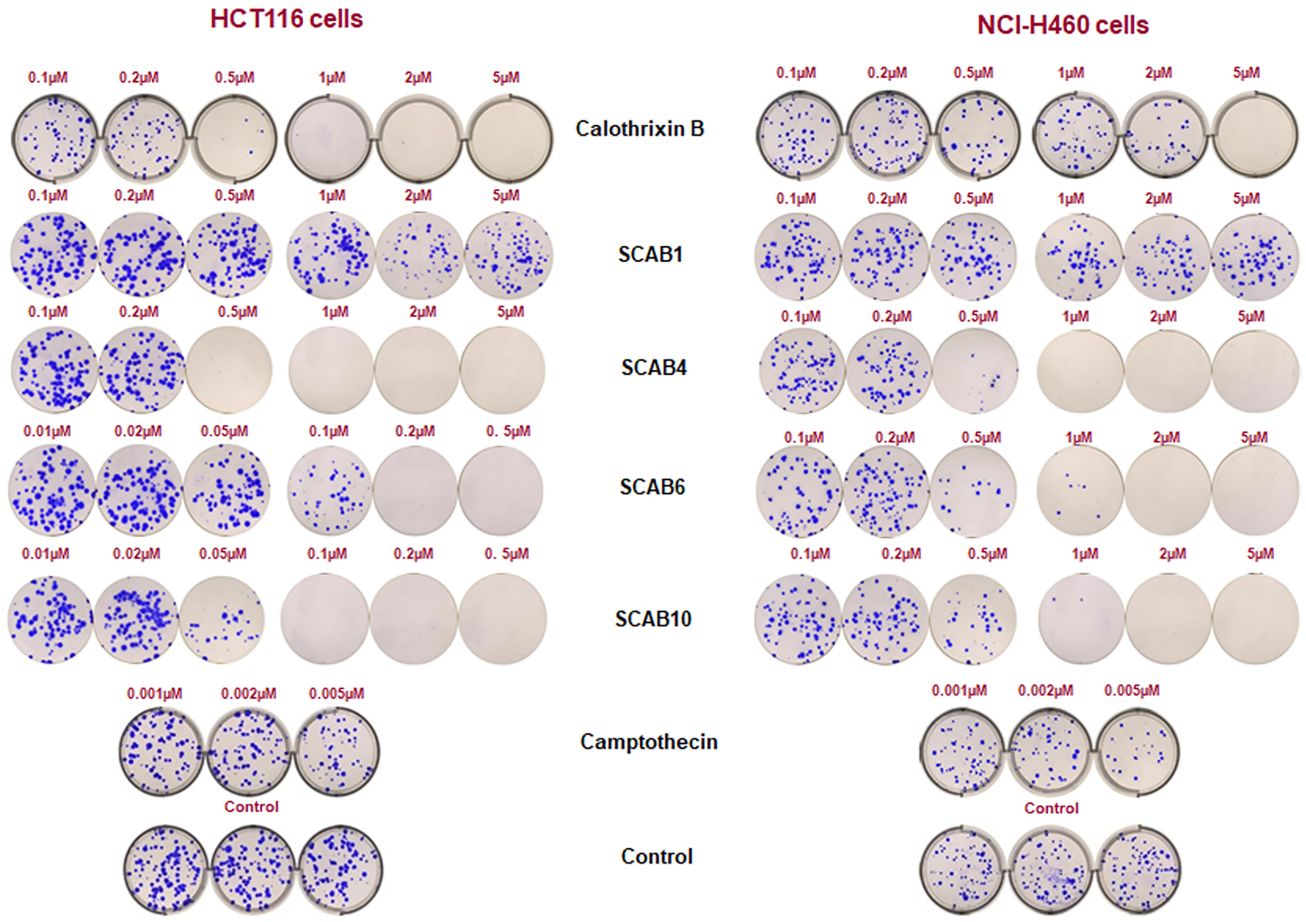
Isothiacalothrixins severely affected the clonogenicity of HCT116 and NCI-H460 cells. The effects of calothrixin B, thiacalothrixin B and isothiacalothrixin analogues on the clonogenic growth of colon adenocarcinoma HCT116 and NCI-H460 cell lines. Cells were seeded in a six-well plate and after over-night adherence, treated with different concentrations (0.1–5 μM) of calothrixin B, thiacalothrixin B and isothiacalothrixins (**SCAB 4**, **6** and **10**), for 48 hours. After drug treatment, the cells were washed with Dulbecco’s phosphate buffered saline and let grow up to 14 days in drug-free medium. Cell colonies were stained with crystal violet and photographed.

The long-term effect of thia analogues of calothrixin B on permanent cell growth arrest and cell death was assessed through clonogenic survival assays. The isothiacalothrixin B analogues inhibited the clonal growth of HCT116 and NCI-H460 cells in a dose-dependent manner as shown in Fig 3 and S2 Fig. Further isothia analogues namely, **SCAB 4**, **6** and **10** abolished the clonal growth of HCT116 cells in a way similar to that of calothrixin B on the other hand; these analogues could inhibit the colony formation of NCI-H460 cells which are practically resistant to calothrixin B (Fig 3). Conversely, the thia analogue **SCAB 1** was not able to abolish colony formation even at the higher concentration of 5 μM taken for the study (Fig 3).

### Isothiacalothrixins induces cell cycle arrest in HCT116 cells

**Fig 4:**
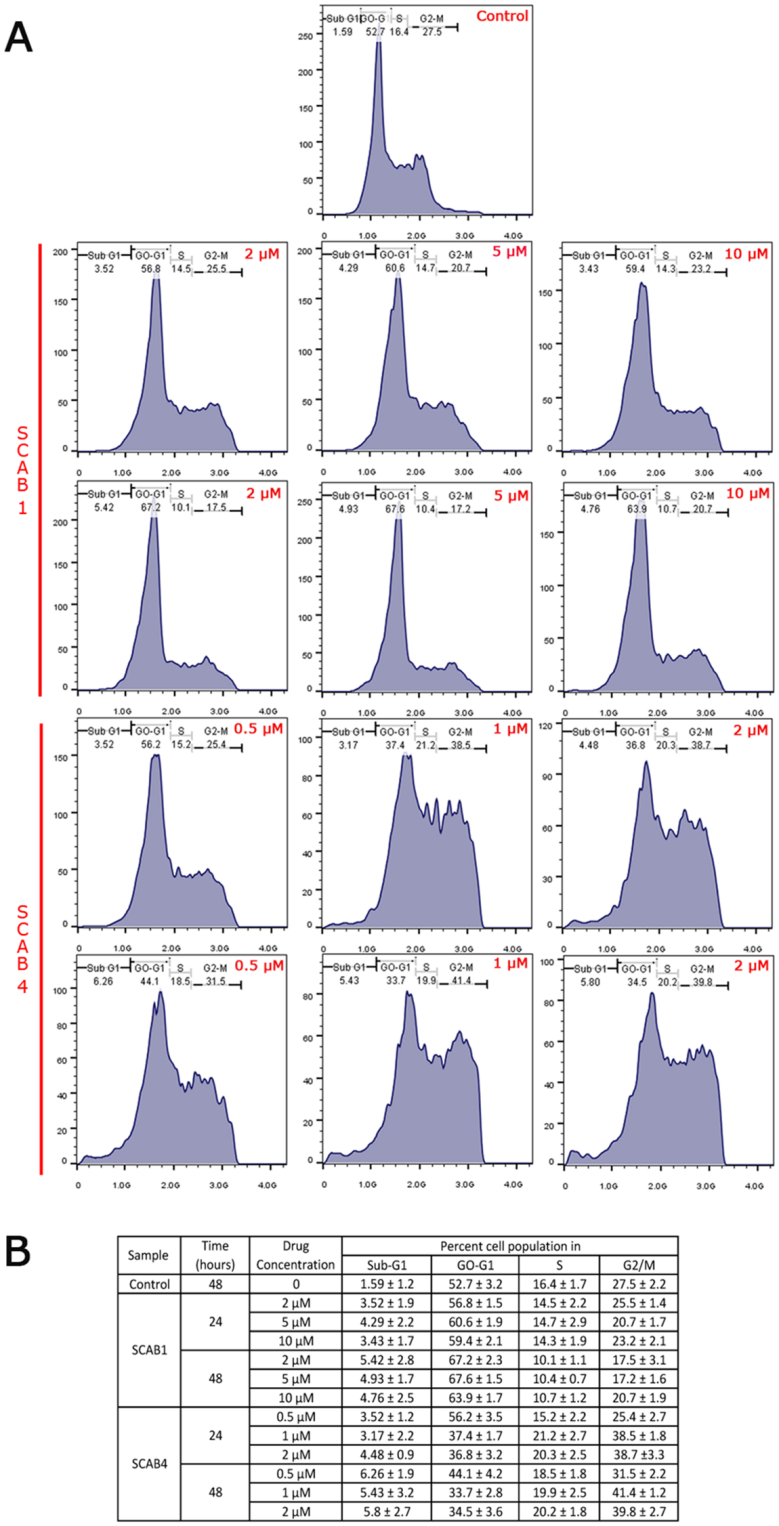
Isothiacalothrixin B (SCAB 4) analogues caused time- and dose-dependent arrest of cells in S and G2/M phases. (A) Cell cycle analysis of human colon HCT116 cells treated with different concentrations (1, 2 and 5 μM) of the thiacalothrixins B (**SCAB 1**) or (0.5, 1 and 2 μM) isothiacalothrixins B (**SCAB 4**) for 24 and 48 hours. (B) The table shows the Mean ± SD percentage of cells in the G0/G1, S and G2/M phases of the cell cycle. Figures are representative of other two experiments.

**Fig 5:**
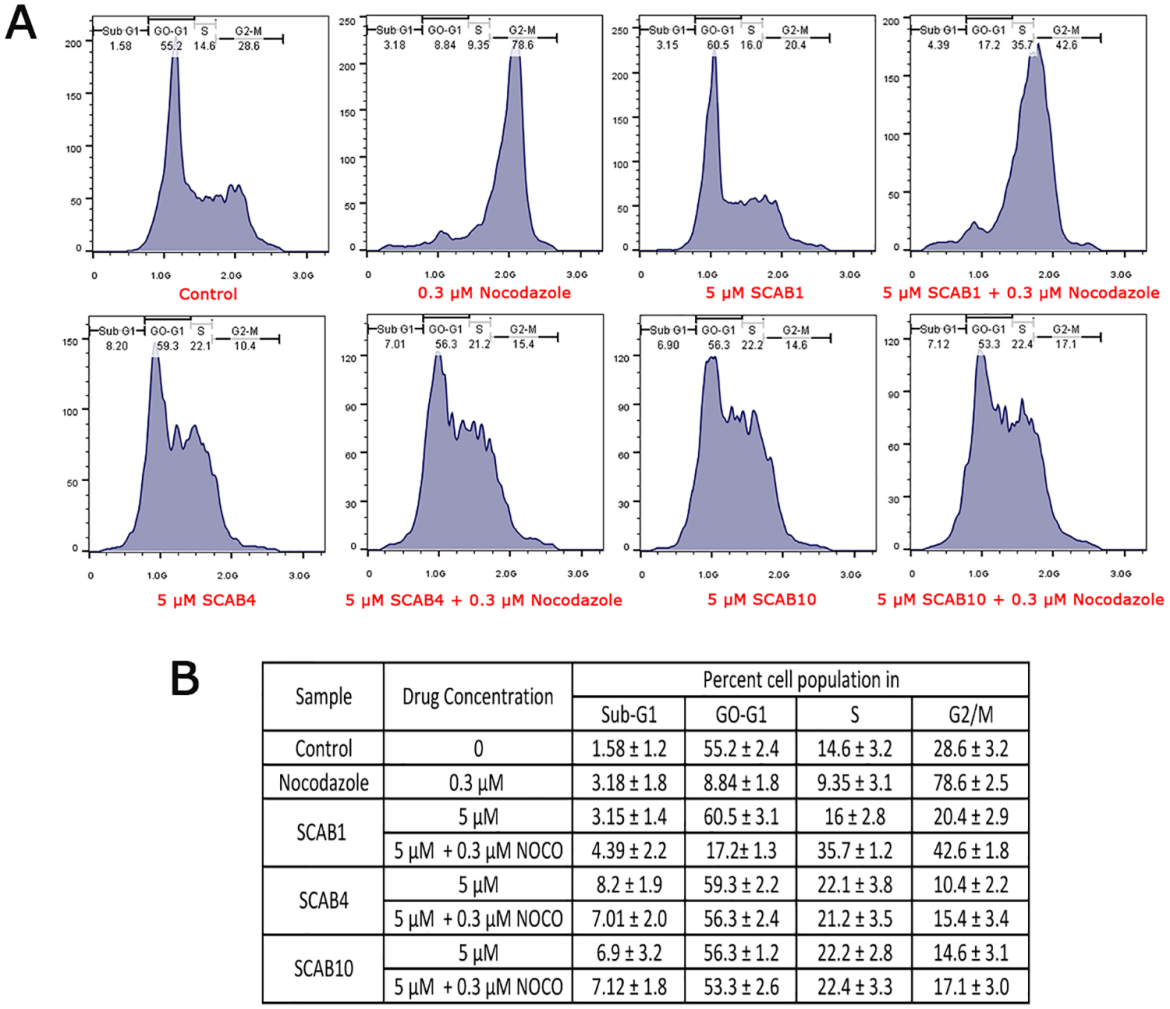
Isothiacalothrixin B (SCAB 4) but not thiacalothrixin B caused the arrest of cells in S and G2/M phases. Cell cycle perturbation by thiacalothrixin B (**SCAB 1**) or isothiacalothrixin B (**SCAB 4, SCAB 10**) in the presence or absence of nocodazole. HCT116 cells were treated with 5 μM of thiacalothrixin B and its analogues for 20 hours or 3 hours pretreatment with 17 hours in the presence of nocodazole followed by propidium iodide staining. The population of cells in different phases of the cell cycle were analysed by flow cytometry. (B) The table shows the Mean ± SD percentage of cells in the G0/G1, S and G2/M phases of the cell cycle. Figures are representative of other two experiments.

The distribution of cells in different phases of the cell cycle after treatment of HCT116 cells with thia calothrixin B analogues at a different dose and time points was analysed with flow cytometry and propidium iodide (PI) staining. The result showed that thia calothrixin B induced only a marginal increase in G0/G1 arrest in a time-dependent manner even at a higher dose of 10 μM (data not shown). On the other hand, isothia analogue **SCAB 4** caused an accumulation of cells in S and G2/M phase which was maintained over 48 hours of drug treatment. As shown in Fig 4A and 4B, **SCAB 4** at concentrations of 0.5, 1, and 2 μM induced a remarkable increase in cell populations in both S and G2/M phase. To identify the phase of the cell cycle, which were most affected by the action of **SCAB 4** and **10**, the mitotic inhibitor, nocodazole was added to the culture plates following treatment with thiacalothrixin B analogues. The nocodazole treatment was used to prevent the cycling cells to re-enter G0/G1 phase, which was eventually getting arrested in mitotic phase. Fig 5, shows that no cell cycle effect (arrest) was observed with 5 μM of thia calothrixin (**SCAB 1**) treatment to HCT116 cells, where all the cycling cells got trapped in mitotic phase following nocodazole treatment. However, in the case of isothia analogues (**SCAB 4** and **10**), the cells were getting truly arrested in S and G2/M phase and were unaffected by subsequent nocodazole treatment (Fig 5A and 5B). Thus, it is evident from cell cycle analysis that isothiacalothrixin B analogues treatment resulted in the accumulation of cells arrested in S and G2/M phase with a concurrent decrease in the proportion of cell in the G0/G1 phase.

### Isothia analogues directly damage DNA in the presence of a reductant

**Fig 6:**
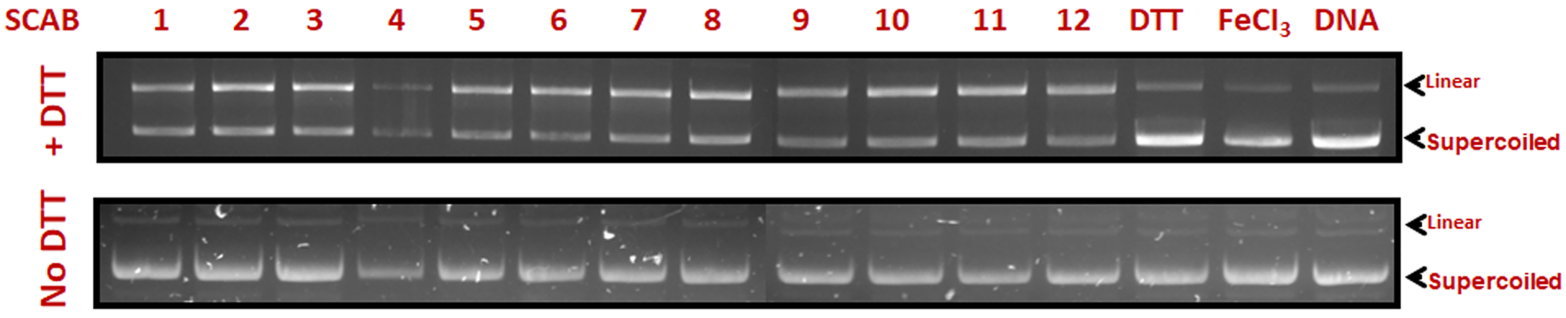
Thiacalothrixin B and its analogues cleaved plasmid DNA in a cell-free system. Supercoiled plasmid DNA was incubated for 1 hour with 250 μM of thiacalothrixin B analogues, 200 μM of ferric chloride in the presence or absence of DTT (200 μM). Plasmid DNA was separated by agarose gel and stained with ethidium bromide.

Gel electrophoresis experiments were performed to monitor the cleavage of pBSK (+) plasmid DNA when incubated with thiacalothrixins in the presence or absence of metal ion (Fe^3+^ ion) and dithiothreitol (DTT) as a reductant. The experimental results were shown in Fig 6. A clear single band was observed for the controls which represent the relatively fast migrating intact super coil form (SC). The second slow-moving band seen in the drug-treated lanes represents the open circular or linear form (RL) which was generated from supercoiled when scission occurred in one of its strands. There was a significant increase in the amount of linear form following treatment with thiacalothrixins, the doubly substituted (both A and E ring substituted isothiacalothrixins) analogues; there was a more significant increase in the linear form (> 65%). It was evident that the isothiacalothrixins have more ability to induce strand cleavage of supercoiled plasmid DNA (S3 Fig). An interesting observation to note with the isothiacalothrixin **SCAB 4** was a reduction in the band intensities of both linear and supercoiled form indicating that this analogue might possess nuclease activity which results in degradation of plasmid DNA even in the absence of reducing agent (Fig 6, lane 4).

### Thia analogues of calothrixin B induces DNA strand breaks

**Fig 7:**
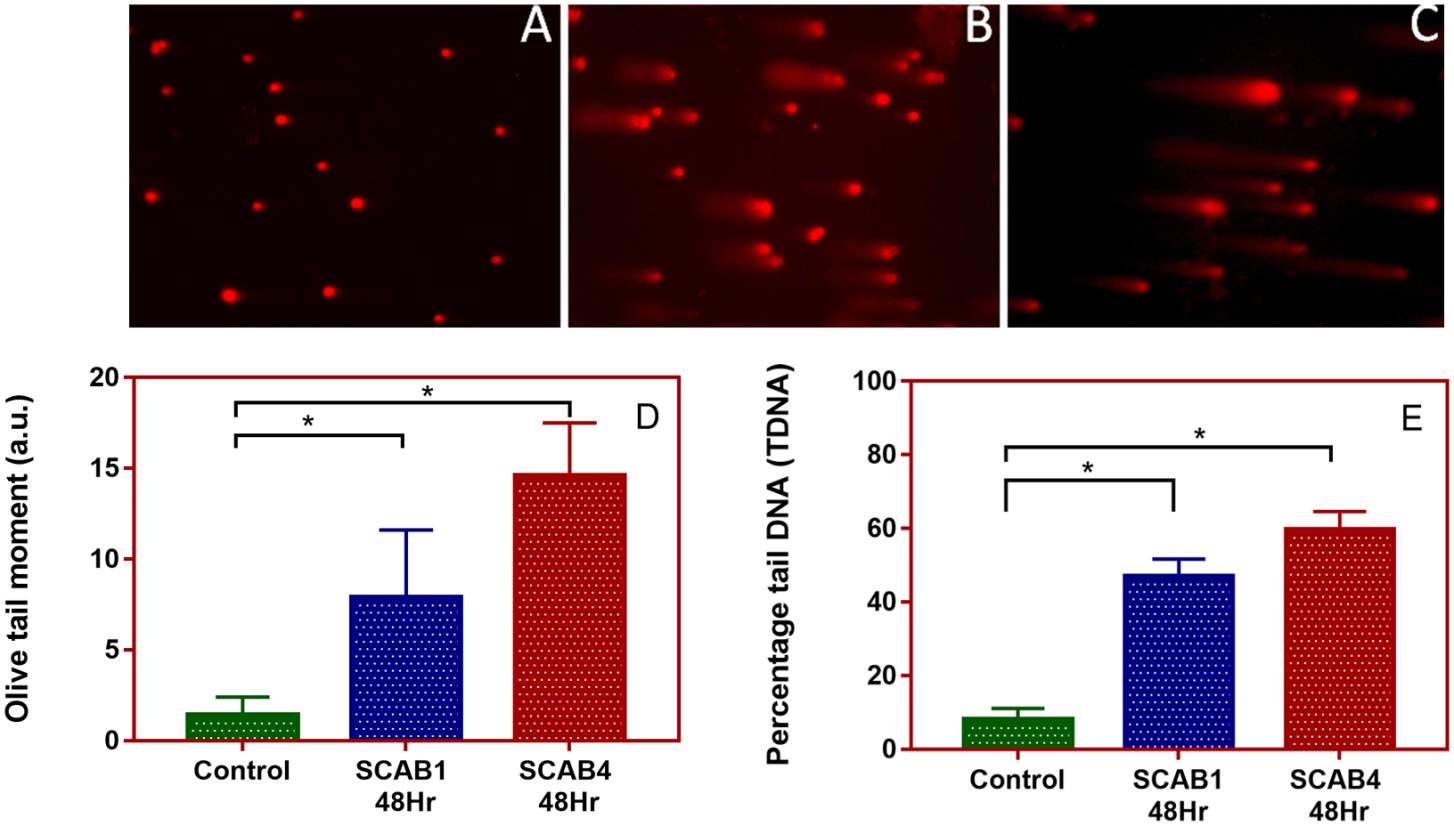
Thia analogues of calothrixin B analogue induced DNA strand breaks. Single-cell gel electrophoresis data (comet assay) in HCT116 cells (A) treated for 48 hours with 5 μM thiacalothrixin B, **SCAB 1** (B) or 5 μM isothiacalothrixin B, **SCAB 4** (C). Olive tail moment (D) and mean percentage tail DNA (E) in HCT116 cells exposed to 5 μM of SCAB1 or SCAB4 for 48 hours. Values are represented as Mean ± standard deviation; *, *P < 0*.*05*. Experiments were performed in duplicate.

The ability of the synthesised thia analogues to induce DNA strand breaks were examined in mammalian cells. The alkaline COMET assay was used to detect DNA damage in the form of single-strand breaks and alkali-labile sites after exposure to 5 μM of thia/isothiacalothrixin B analogues for 48 hours to HCT116 cells. The alkaline comet assay is simple and known for its sensitivity to detect DNA strand breaks [27]. The single cell suspension obtained from drug-treated culture plates were embedded in agarose on a microscope slide, lysed and electrophoresed at a high pH (>13). The nucleoid with broken DNA is drawn towards the anode, thus appearing as a ‘comet tail’ following staining with ethidium bromide and observed under fluorescence microscope. The images of HCT116 cells treated with thiacalothrixins or calothrixin B showed the formation of comets whereas no comet pattern was found in control cells (Fig 7). However, the isothiacalothrixin **SCAB 4**, caused profound DNA strand breaks as evident from the significant increase in olive tail moment (OTM) and percentage tail DNA (TDNA) (Fig 7D and 7E) in comparison to its parent compound, **SCAB 1**. Also, the calothrixin B did not exhibit DNA strand breaks to the extent of isothiacalothrixin (data not shown). The increase in DNA damage suggested that isothiacalothrixin induced fragmentation of chromosomal DNA leading to apoptotic cell death. Further, the results from DNA cleavage in a plasmid DNA-based assay was complemented with the more direct DNA strand breaks in cellular milieu by comet assay.

### DNA binding mode and affinities of thiacalothrixin analogues

Many clinically useful small molecule anticancer agents with accessibility to chromosomal DNA can bind with genomic DNA [28]. The low molecular weight calothrixins and its thia analogues often carrying charges resulting in its interaction with DNA. To understand the DNA interaction with the planar aromatic quinone containing calothrixins/thiacalothrixins, UV-Vis spectroscopic studies were carried out with representative calothrixins with calf-thymus DNA (CT-DNA). The UV-Vis spectra of the calothrixin B or isothiacalothrixin B, whose structures are given in Fig 1, are characterized by moderate absorption in the visible regions at 285 and 369 nm (S4 Fig). However, upon addition of varying concentrations of CT-DNA to a fixed amount of calothrixins resulted in little hypochromic shift (4%) without any band shift, indicating groove interaction between the charged calothrixin species with the bases of DNA (S4 Fig).

*In silico* molecular modelling studies with the DNA and calothrixins was carried out to get some insight on the interaction between them and to corroborate the experiment results. The docking studies of calothrixins with the 3D structure of 1DSC DNA was determined and the one having lowest binding energy among the different conformations were generated. The binding energy value obtained from docking interactions is suggestive of the strength of drug-DNA interaction, where lower the binding energy signifies higher the binding affinity and *vice versa* [29]. The Autodock conformations for calothrixins indicated a lower binding energy values calothrixin B followed by A and E or A/E halo substituted isothiacalothrixin B analogues whereas thiacalothrixin B was computed to have higher binding energy value (Table 2). The calothrixin B has a highest binding affinity for 1DSC DNA (binding energy value = -7.2 kcal/mol), whereas binding energy values for isothiacalothrixin B (**SCAB 4**) and thiacalothrixin B (**SCAB 1**) were -6.13 kcal/mol and -5.49 kcal/mol, respectively.

**Table 2:** *In silico* calculations of free binding energy scores for the calothrixin B and thiacalothrixin B analogues ligands docked with octamer DNA.

S.NO	Name of the compound	BINDING ENERGY	INTERACTING BASE PAIRS WITH DYE
1	CAB1	-7.2	5’ *GP*AP*AP*GP*CP*TP*TP*C 3’
2	SCAB8	-6.71	5’ *GP*AP*AP*GP*CP*TP*TP*C 3’
3	SCAB7	-6.48	5’ *GP*AP*AP*GP*CP*TP*TP*C 3’
4	SCAB12	-6.28	5’ *GP*AP*AP*GP*CP*TP*TP*C 3’
5	SCAB11	-6.19	5’ *GP*AP*AP*GP*CP*TP*TP*C 3’
6	SCAB6	-6.17	5’ *GP*AP*AP*GP*CP*TP*TP*C 3’
7	SCAB9	-6.16	5’ *GP*AP*AP*GP*CP*TP*TP*C 3’
8	SCAB4	-6.13	5’ *GP*AP*AP*GP*CP*TP*TP*C 3’
9	SCAB2	-6.06	5’ *GP*AP*AP*GP*CP*TP*TP*C 3’
10	SCAB5	-6.04	5’ *GP*AP*AP*GP*CP*TP*TP*C 3’
11	SCAB10	-6.01	5’ *GP*AP*AP*GP*CP*TP*TP*C 3’
12	SCAB1	-5.49	5’ *GP*AP*AP*GP*CP*TP*TP*C 3’
13	SCAB3	-5.35	5’ *GP*AP*AP*GP*CP*TP*TP*C 3’

The different isomeric forms of DNA arise due to its inherent flexibility [30, 31] and choosing the appropriate oligomer conformation for docking studies remains a significant question, mainly when the mode of binding of a ligand to DNA was not known [32]. Thus, the nature of the chosen oligomer conformation dramatically affects the docking performance when the ligand interacts with DNA through an induced fit [32]. There are two kinds of non-covalent interaction between the drug and the DNA, namely groove recognition (minor or major groove) or intercalation between the base pairs [33]. In general, most of the small molecules prefer to bind to the minor groove although the major groove offers a larger and shallow binding pocket with the more H-bonding donor as well as acceptor sites. The planar aromatic molecules such as ellipticine prefer an intercalative binding wherein structural changes to DNA (unwinding and lengthening) happens and is contemplated as an induced fit mechanism [32].

**Fig 8:**
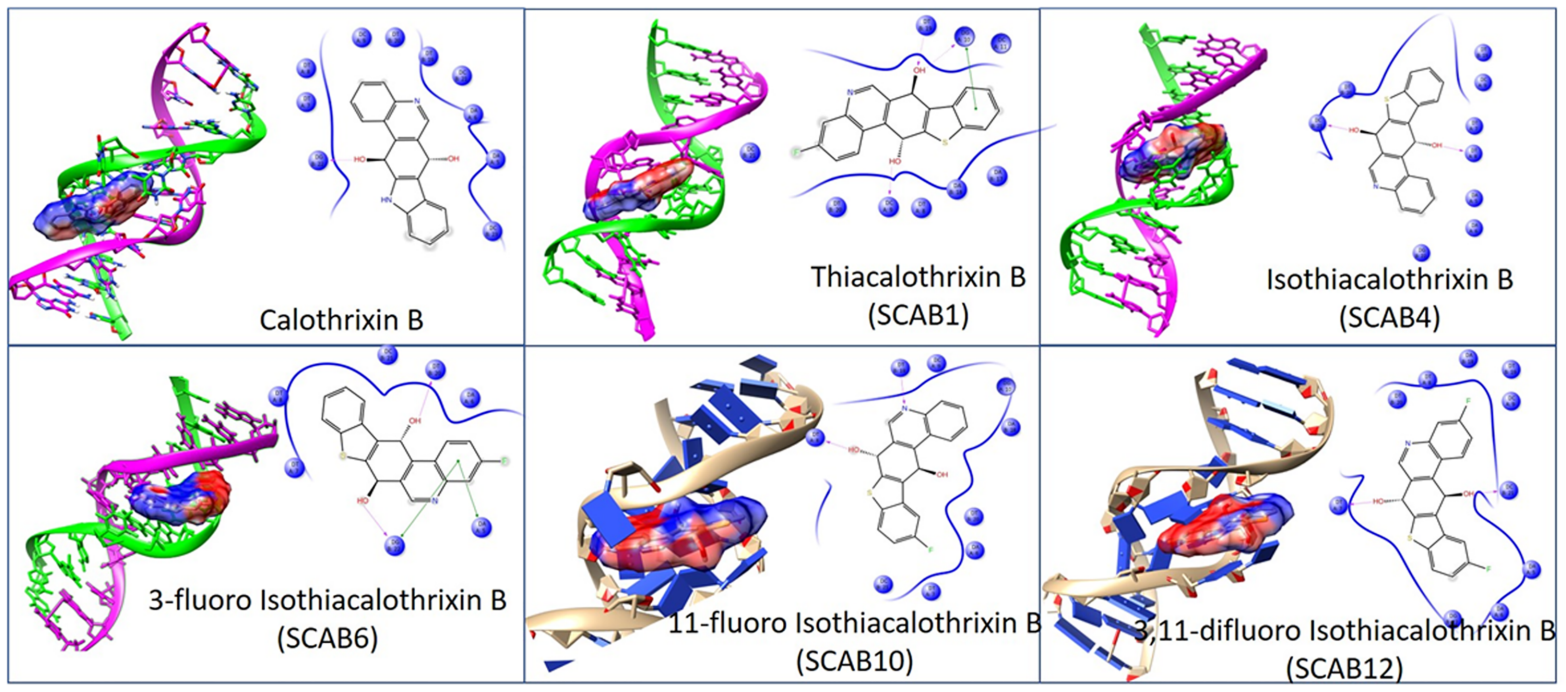
The isothiacalothrixin B analogues fit deep into the minor groove of DNA. Best docking conformations of calothrixin B and its thia analogues with crystallographic DNA (PDB ID: 453D DNA) towards left and a two-dimensional representation of key structural moieties along with their base-pair interactions depicted on to the right.

In our molecular modelling studies minor groove recognition by calothrixin and its thia analogues have made a fit, like a lock- and key- mechanism. Due to their flexibility, the isothiacalothrixin derivatives like **SCAB 4**, **6, 10** and **12**, assume a curved shape that matches DNA topology and allows a comfortable fit of the ligand in the minor groove (Fig 8). Different binding poses of calothrixins and its analogues and their residual interactions between base pairs of DNA (PDB ID: 453D [21]) have been shown in Fig 8. Main factors which govern this type of interaction depends upon factors viz., van der Waals contacts playing pivotal role reinforced by hydrogen bonding and electrostatic interactions. Moreover, the selective binding mechanism of the calothrixins and its thia analogues to AT-rich sequences in the minor groove, indicate that it is possible to achieve sequence-selective binding agents. As shown in Fig 8, for instance, analogues like **SCAB 4**, **6** & **SCAB 10** did interact strongly with AT-rich sequences, displaying large residence times. Concerning the thiacalothrixin B analogues the binding free energies of the isothia analogues, were comparable, with a very favourable average binding energy of -7.18 kcal/mol for DNA (PDB ID: 453D) Table 3.

**Table 3:** Docking results of calothrixin B and its thia analogues with DNA (PDB ID: 453D).

Compound No	ΔG (Kcal/mol)	Glide Energy (Kcal/mol)
**SCAB 6**	**-7.05**	**-46.22**
**SCAB 4**	**-8.35**	**-48.10**
**SCAB 12**	**-6.73**	**-43.56**
**SCAB 10**	**-6.59**	**-46.90**
**SCAB3**	**-6.19**	**-48.88**
**CAB 1**	**-5.58**	**-48.31**
**SCAB 1**	**-3.85**	**-47.21**

### Calothrixin B or thiacalothrixin B induces p21^waf1/cip1^ but not p53 protein in HCT116 cells

**Fig 9:**
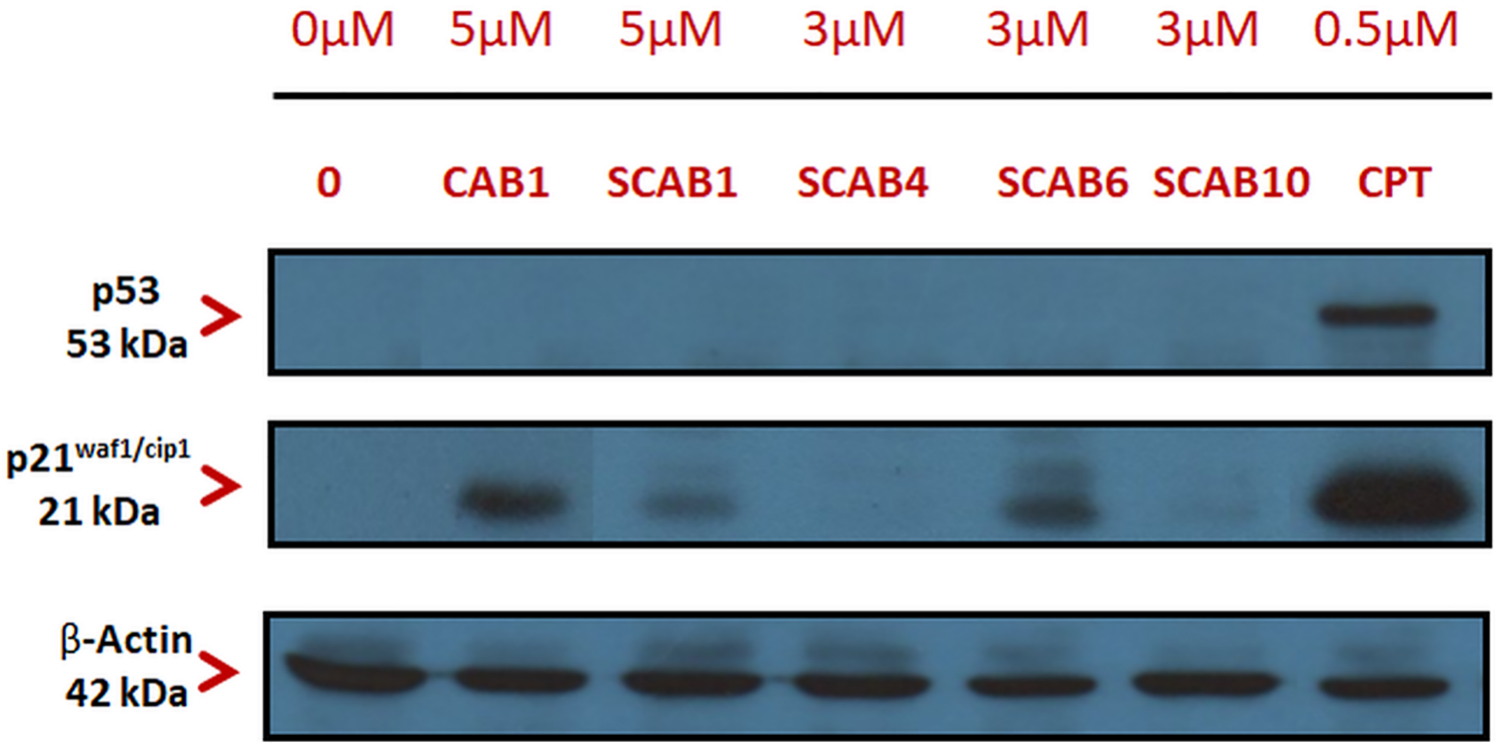
Calothrixin B or thiacalothrixin B but not isothiacalothrixins induced p21 with no upregulation of p53. Western blot analysis of p53 and p21^waf1/cip1^ protein in HCT116 cells after treatment without or with 5 μM thiacalothrixin B analogues for 48 hours. The whole cell lysate was immunoblotted with p53 and p21 antibody, respectively. The same blot was re-probed with β-actin to confirm equal loading of each lane.

To determine whether the cytotoxic effect of thia calothrixin B analogues in HCT116 cells was analogous to its activation in response to DNA damage, we assessed the protein levels of p53, a tumour suppressor and p21^waf1/cip1^, a CDK inhibitor which are involved in cell cycle arrest and apoptosis. Western blotting analysis revealed no upregulation of p53 protein (Fig 9) even in the case of isothiacalothrixin treated HCT-116 cells which showed signs of DNA damage in COMET assay. In contrary, the CDK inhibitor p21^waf1/cip1^ was upregulated in Calothrixin B, and its thia analogue **SCAB 1** treated cells but not with the isothia calothrixins taken for the study (Fig 9).

### Calothrixin B or thiacalothrixin B activates p21^waf1/cip1^ promoter by a p53- independent mechanism

**Fig 10:**
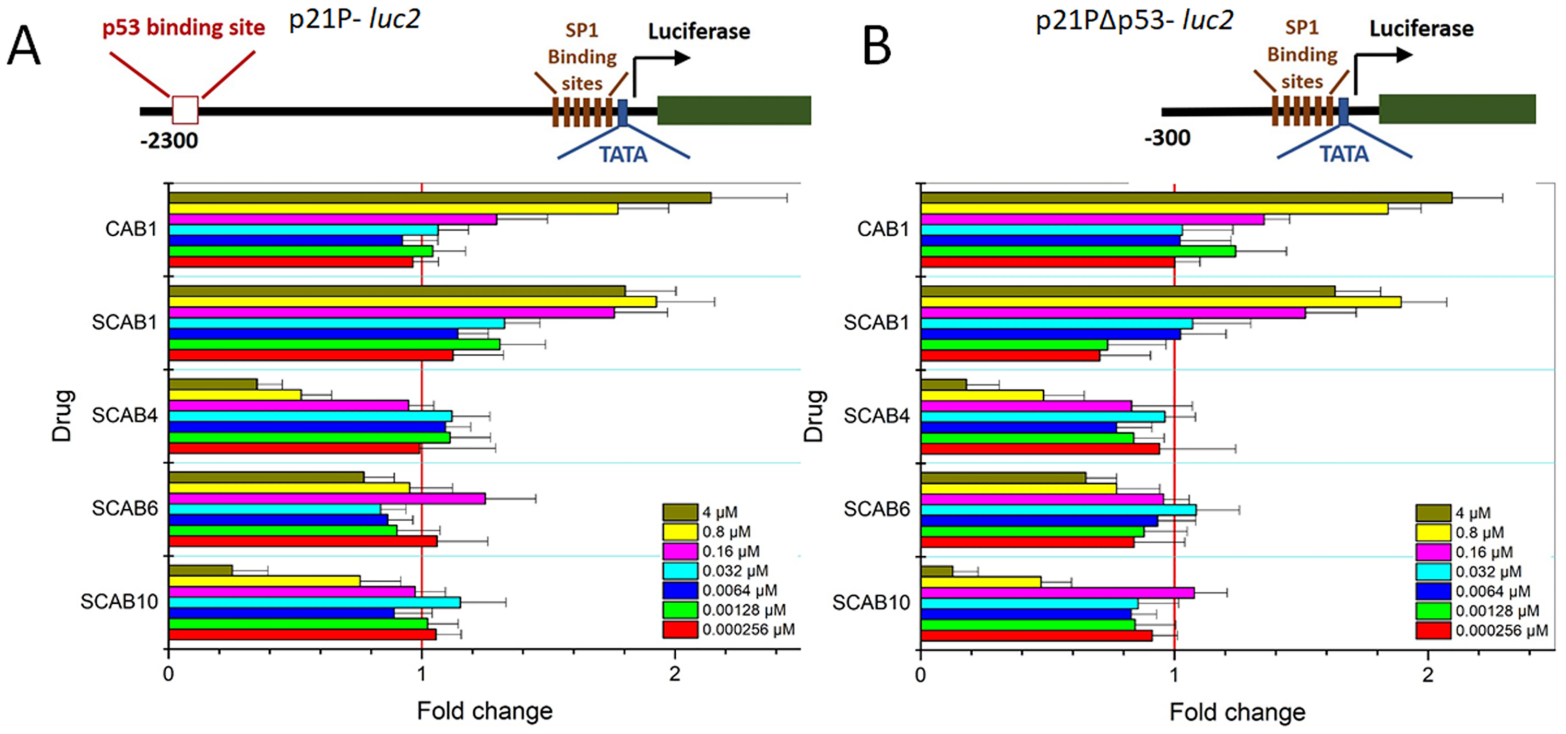
Transcriptional activation of p21 by calothrixin B or thiacalothrixin B independent of p53. HCT116 cells were transfected with the wild-type (p21P-*luc2*) or the truncated p21 (p21PΔp53-*luc2*) promoter region, exposed to different concentrations of calothrixin B or thicalothrixin B analogues for 48 hours, then harvested and assayed for luciferase activity. Histograms represented firefly luciferase activity in each sample and expressed as fold induction relative to controls for each reporter, of which the values were taken as 1. All the values are the average of triplicate samples from a typical experiment; *bars*, ±SE.

To investigate whether the upregulation of p21^waf1/cip1^ protein by calothrixins is due to transcriptional activation of the p21^waf1/cip1^ promoter, a plasmid construct harbouring reporter a luciferase reporter gene under the transcriptional control of wild-type (p21P-*luc2*) and truncated (p21PΔp53-*luc2*) p21^waf1/cip1^ promoter, was transfected into HCT116 cells and calothrixin induced luciferase activity was measured. Sixteen hours after transfection, HCT116 cells were exposed to different concentrations of calothrixins for 48 hours, and luciferase activity was measured as described in “Experimental section”. The transcriptional activity of the full length (-2300/+8) p21^waf1/cip1^ promoter was significantly increased in a dose-dependent manner only in the calothrixin B/thia calothrixin B treated cells (Fig 10A). The isothaicalothrixin analogues failed to activate the full-length p21^waf1/cip1^ promoter at all the concentrations tested. Further to determine whether the transcriptional activation of p21^waf1/cip1^ by calothrixin B or its thia analogue **SCAB 1**, the HCT116 cells were transfected with a p21PΔp53-*luc2* promoter (-300/+8) which lacks the p53 consensus binding site present towards the 5’end. The activation profile of p21PΔp53-*luc2* promoter was similar to the full-length p21^waf1/cip1^ promoter (Fig 10B) indicating that the presence of p53 binding sites in the p21 promoter was not required for calothrixins/thiacalothrixins to activate the p21^waf1/cip1^ promoter.

### Morphologic evidence for isothiacalothrixin induced apoptosis

**Fig 11:**
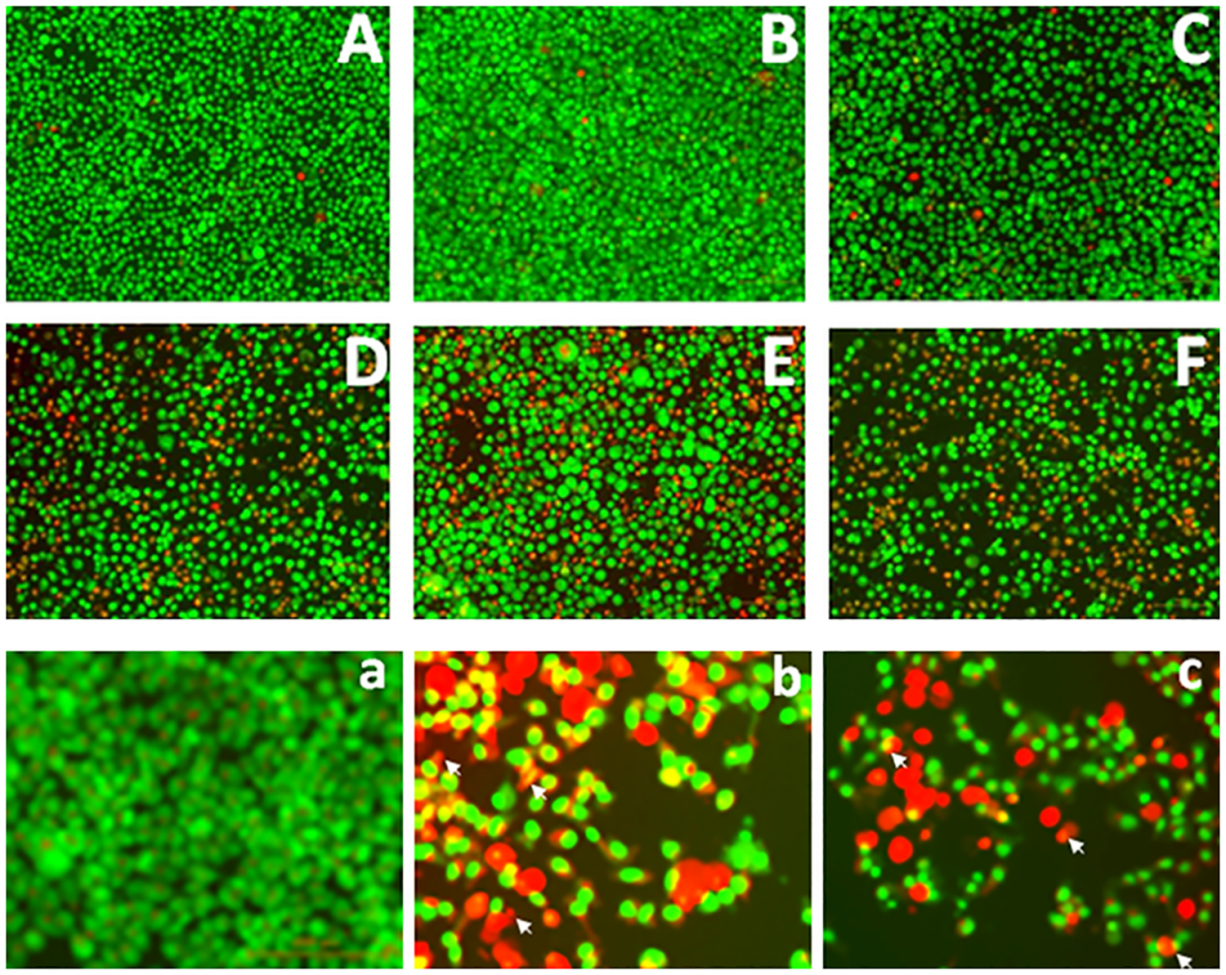
Isothiacalothrixin B analogues induced apoptotic morphology. HCT116 cells were stained with acridine orange/ethidium bromide after 48 hours of treatment with calothrixin B/ thiacalothrixin analogues. Cells were observed under fluorescence microscope (x100 magnification). Viable cells show green fluorescence. Necrotic and apoptotic cells show orange and yellow fluorescence. (A) Untreated HCT116 control cells. Cells were treated with 5 μM of calothrixin B, **CAB 1** (B) 5 μM of thiacalothrixin B, **SCAB 1** (C), 5 μM of isothiacalothrixin B **SCAB 4** (D), 5 μM of 3-Fluoro isothiacalothrixin B, **SCAB 6** (E), 5 μM of 11-fluoro isothiacalothrixin B, **SCAB 10** (F). Cells were observed under fluorescence microscope (x400 magnification), (a) Untreated HCT116 control cells. Cells were treated with 5 μM of thiacalothrixin B, **SCAB 1** (b), 5 μM of 11-Fluoro isothiacalothrixin B, **SCAB 10** (c).

The presence of a sub-G1 population of cells seen in flow cytometric analysis in thiacalothrixin treated HCT116 cells suggested induction of apoptosis following the arrest of cells in G1-S phase. To confirm this, the cellular morphology of drug-treated cells was observed microscopically following staining of unfixed, non-permeabilized cells with DNA- intercalating dyes acridine orange and ethidium bromide. Acridine orange being cell permeable can enter healthy or dying cells whereas ethidium bromide can only enter dying or dead cells whose membrane integrity is compromised. The results obtained with the AO/EB staining of HCT116 cells treated with thia analogues of calothrixin for 48 hours are shown in Fig 11. The untreated control cells or calothrixin B and its thia congener **SCAB 1** were characterised by a bright green nucleus with uniform intensity (Fig 11A–11C). Whereas, most of the cells treated with isothaia analogues (**SCAB 4, 6 & 10**) were orange-red in appearance (Fig 11D–11F). Fluorescence microscopic images of the isothiacalothrixin treated cells Fig 11b and 11c, clearly revealed morphological alterations like extensive nuclear condensation and fragmentation which are characteristic of apoptotic cells.

### Isothia analogues induce DNA fragmentation

**Fig 12:**
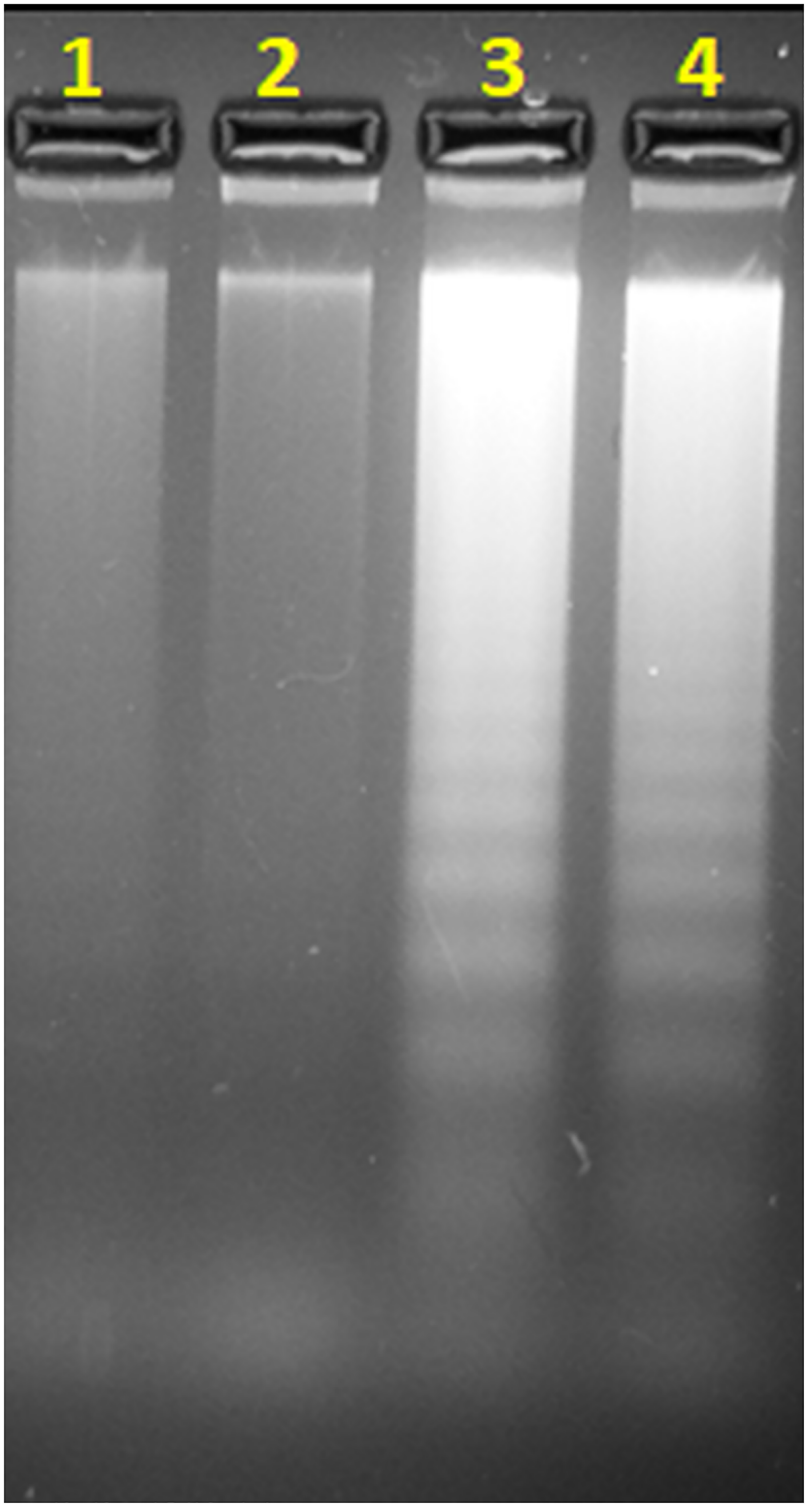
Isothiacalothrixin B analogues induced DNA fragmentation. HCT116 cells were treated with 5 uM of thiacalothrixin B (**SCAB 1**) or isothiacalothrixin analogues (**SCAB 4**, **10**) The cells were processed as described under "Materials and Methods section". The isolated DNA was electrophoresed on TAE agarose gel for 1.5 h at 100 V. The DNA fragments were visualised by staining with ethidium bromide. Lane 1 = DNA from untreated HCT116 cells; lane 2 = DNA from thiacalothrixin B (**SCAB 1**) treated cells; lane 3 = DNA from isothiacalothrixin B (**SCAB 4**) treated cells; lane 4 = DNA from 11-Fluoro isothiacalothrixin B (**SCAB 10**) treated cells.

Internucleosomal DNA fragmentation is a classic signal of apoptosis in mammalian cells. HCT116 cultured with DMSO (control) or with 5μM of thiacalothrixin for 48 hours showed no DNA laddering. However, the cultured HCT116 cells upon treatment with 5μM isothiacalothrixin B analogues (**SCAB 4** and **10**) contained fragmented DNA wherein a ladder of DNA bands due to the cleavage of genomic DNA into the nucleosomal size of 180- to 200-bp fragments was seen (Fig 12 **lane 3, 4**). These results confirm that isothiacalothrixins activated apoptosis in HCT116 cells.

### Cellular effects of isothiacalothrixin B analogues are irreversible

**Fig 13:**
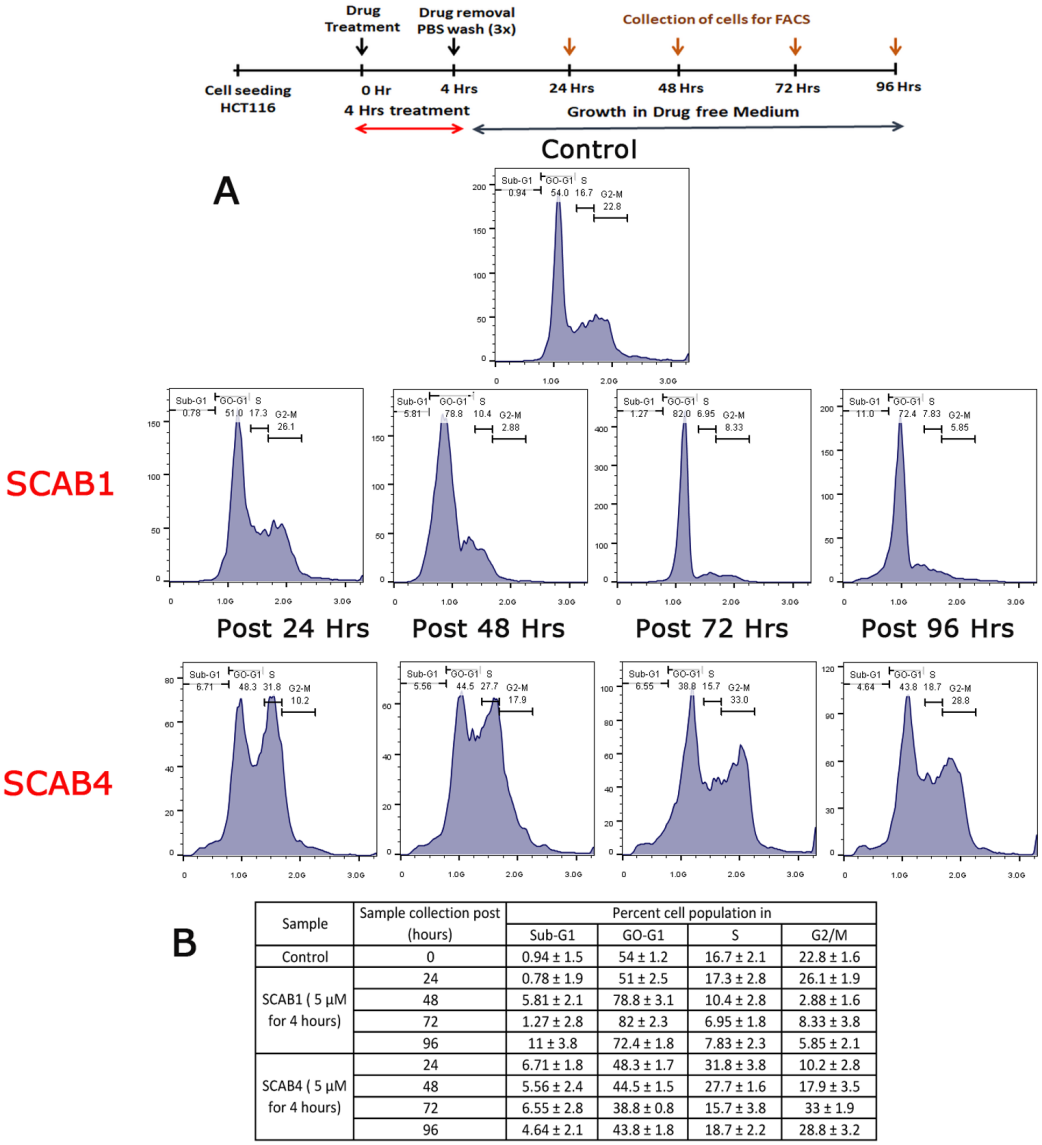
The cell cycle effects of isothiacalothrixin B (SCAB 4) were irreversible. (A) Cell cycle perturbation at 24, 48, 72 and 96 hours post-treatment for 4 hours with thiacalothrixin B (**SCAB 1**) or isothiacalothrixin B (**SCAB 4**). HCT116 cells were treated with 5 μM of thiacalothrixin B and isothiacalothrixin B for 4 h followed by washing with PBS, and collection of cells at 24, 48, 72 and 96 hours in the recovery period and staining with propidium iodide. The percent population of cells in different phases of the cell cycle (B) were analyzed by flow cytometry. The figure is representative of other two experiments.

To check whether the strong growth inhibitory effects of isothiacalothrixins were reversible, HCT116 or NCI-H460 cells were initially exposed to Calothrixin B, thiacalothrixin B or isothiacalothrixin for a brief period, i.e., 4 hours. Following which the cell monolayers were then washed thoroughly to remove the compounds, and the cells were cultured in complete drug-free medium for a further period, i.e., of 24, 48, 72 and 96 hours for flow cytometry analysis, 48 hours for the alkaline COMET assay and 12 days in the case of the clonogenic assay. The cell cycle analysis following four-hour treatment of calothrixin B or thiacalothrixin B resulted in cells reviving to normalcy within 24 hours of growth in drug-free medium whereas arrest of cells in S-G2/M phase were seen until 96 hours after drug removal in the case of isothiacalothrixin B (Fig 13A and 13B). This indicates that the DNA damage produced by isothiacalothrixin was profound and the cells were unable to repair the damaged DNA even until 96 hours after its exposure to the drug for a short time, i.e., 4 hours. Similar results were obtained in the case COMET assay, wherein the isothaicalothrixin but not thiacalothrixin B treated HCT116 cells kept DNA damage until 48 hours after drug removal which was evident from the appearance of comet tails and increase in olive tail moment and percentage tail DNA (S5 Fig). The above results were reflected on the long-term cell survival wherein the clonogenicity of HCT116 cells was affected by short time treatment with isothiacalothrixin B whereas the colony forming potential of thiacalothrixin B/calothrixin B treated cells were unaffected following treatment with 5 μM for 4 hours (S6 Fig).

## Discussion

The natural product calothrixin B and its fluoro analogues have shown potent cytotoxic effects against certain of human cancer cell lines [2]. Thus, considering calothrixin B as a useful lead compound to discover novel chemotherapeutic agents, the sulphur isostere analogues of calothrixin B were synthesised [6]. Our preliminary study of the anticancer spectrum of thia analogues of calothrixin B in various cell lines, identified isothiacalothrixins are having potent anti-tumour activities [6]. Similar studies with ellipticine, an anti-tumour alkaloid structurally related to calothrixin B identified thiaellipticine to be equally active and in contrary isoellipticine was inactive [34] whereas the isothiaellipticine was found to be effective as that of ellipticine. However, several 7-substituted isoellipticines and isoellipticinium salts showed promising anti-cancer activity in NCI-60 screening and inhibition of human topoisomerase II activity [35]. The cellular effects of 7-hydroxyisoellipticine on the Acute Myeloid Leukaemia (AML) cell line, MV4-11 demonstrated the generation of reactive oxygen species (ROS) and subsequent activation of DNA damage pathway to result in the arrest of cells in G2/M phase and apoptosis [36]. Also, another isoellipticine derivative, 7-formyl-10-methylisoellipticine induced ROS mediated cytotoxicity in AML cells and significant reduction of tumour burden in an AML xenograft mouse model [37]. Although isothiacalothrixin has been reported to inhibit cancer cell growth in sub-micromolar concentrations, no information concerning the mode of cell killing has been reported. Thus, this study is the first to demonstrate the cellular effects of thia calothrixin B and its isomer isothiacalothrixin B in HCT116 and NCI-H460, a colon and lung cancer cell lines, respectively. The *in vitro* cytotoxicity assay across six cell lines showed that isothiacalothrixin B analogues were superior in inhibiting the proliferation of cancer cells in comparison to thiacalothrixin B, which itself was less active than the parent analogue, calothrixin B. Also, treatment with 5μM of isothiacalothrixin B analogues to HCT116 cells showed time-dependent increase in dead cells which is not prominent upon similar treatment with thiacalothrixin B. Furthermore, a clonogenic assay was performed to assess the long-term growth effects of thia analogues of calothrixin B. The clonogenic assay which has proven predictive value in chemosensitivity test of cytotoxic compounds, correlated well with the results from SRB assay. Our results demonstrate a concentration-dependent inhibition of clonogenicity in isothiacalothrixin B treated HCT116 and NCI-H460 cells, suggesting that isothiacalothrixins have a therapeutic potential against the colon and lung cancer.

One of the common mechanisms by which the anti-tumour agents exerts its effect is through the induction of cell cycle arrest and apoptosis. It has been reported that fluoro analogue of calothrixin B could cause the arrest of cells in the G1-S phase of cell cycle and apoptosis in HCT116 cells [2]. We analysed the cell cycle effects of thiacalothrixin B in the presence of the mitotic inhibitor, nocodazole to ascertain the truly affected population of cells in the cell cycle. Treatment for 16 hours with nocodazole alone resulted in 70% of the cells progressed across the cell cycle and arrested in G2/M phase. However, administration of isothiacalothrixins little prior (4 hours) to nocodazole resulted in a decrease in the percentage of cells that reached G2/M phase. This reduction in G2/M cells was due to arrest of cells in the G1-S phase of cell cycle whereas thiacalothrixin B treatment to HCT116 cells caused the intermittent G1 arrest of cells which progressed through the cell cycle to get arrested in G2/M phase because of nocodazole treatment. The results from cell cycle analysis indicate the important sign of damage to the cellular DNA following treatment with isothiacalothrixin B but not with thiacalothrixin B which led to the permanent arrest of cells in G1-S phase.

Literature evidence suggests that DNA damage following treatment with cytotoxic agents results in the arrest of cells in S or later in G2/M phase, thus allowing time for the cells to repair damaged DNA [38, 39]. We first demonstrated the ability of isothiacalothrixin B analogues to induce plasmid DNA strand breaks in the presence of metal ion and a reducing agent, dithiothreitol. At the cellular level COMET assay has become one of the most commonly used methods for studying DNA damage [40]. To check for DNA strand breaks, single- cell gel electrophoresis assay (COMET assay) was performed. The results showed that there was a significant DNA strand break in isothiacalothrixin treated HCT116 cells which was evident from the appearance of comet tails and increase in percentage tail DNA. However, the extent of comet tailing was not prominent among the thiacalothrixin B treated cells. Thus, an underlying mechanism of isothiacalothrixin B induced S, G2/M arrest in HCT116 cells seems to be closely associated with its effect on the integrity of DNA.

There are two major modes of drug binding to DNA which include either intercalation between adjacent base pairs and interaction into the minor and major groove. Significant deformation of the DNA to create a binding cavity is required for intercalation whereas minor groove does not require major changes in DNA conformation [41]. We have previously reported that the absence of quinone unit in calothrixin B as in the case of quinocarbazloes favoured intercalation with DNA [2]. *In silico* studies with quinone containing calothrixin B or its thia analogues indicated minor groove binding to the DNA with higher affinity to AT-rich sequences which was confirmed through UV-Vis spectral studies. The overall shape of the isothiacalothrixins resembles that of typical minor groove binders where the presence of quinone unit favours hydrogen bonding interaction with the AT-rich regions of the DNA. It might also be possible that calothrixins in general target AT-rich regions of the DNA sequence and therefore inhibit transcription and expression of specific genes.

Interestingly, the sustained antiproliferative effect of thiacalothrixins was not dependent on p53 as some of the cell lines tested (HeLa, SiHa, MDA-MB231 and U251) lack functional p53. This has prompted us to examine the role of p53 if any, on the relevant aspects of the thiacalothrixin B induced DNA damage response of HCT116 cells (which has functionally active p53). The p53 pathway often gets activated following DNA damage leading to arrest of cells in G1 or G2/M phase [42]. p21^waf1/cip1^ is thought to be an effector for p53-mediated inhibition of cell proliferation in response to DNA damage [26]. Western blot analysis was employed to investigate the protein levels of p53 and p21^waf1/cip1^ following treatment with thiacalothrixin analogues. As expected, upregulation of p53 protein level was not seen with any of the calothrixin analogues taken for the study, whereas the positive control camptothecin, a DNA damaging drug-induced p53 levels. Surprisingly, the p21^waf1/cip1^ protein levels were elevated following treatment with thiacalothrixin B and its parent analogue calothrixin B but not with the isothiacalothrixin analogues. Thus, taking in to account on the DNA damage induced by isothiacalothrixin B and subsequent arrest of cells in G1 and G2/M phase, without an increase in the protein levels of p53 strongly support the notion that the induction of cell death is p53- independent. Several studies have highlighted the p53-independent induction of cell death following DNA damage mediated through the pro-apoptotic p73 [43, 44] or NF-kB (nuclear factor-kB) [45, 46] or by the degradation of anti-apoptotic protein BCL2 [47], and thus it is possible that isothiacalothrixin B analogues caused cell death through any of these mechanisms. Both the thiacalothrixin B and calothrixin B induced accumulation of p21^waf1/cip1^ protein. Studies with full length and deletion mutant p21^waf1/cip1^ luciferase reporter indicated a significant activation of full-length (-2300/8+) and the plasmid construct lacking p53-binding sites (-300/+8), apparently suggesting the transcriptional activation of p21^waf1/cip1^ by thiacalothrixin/calothrixin B was independent of p53 function. Further, the minimal p21^waf1/cip1^ promoter required by thiacalothrixin B is similar to the region modulated by Ras, highlighting the possible involvement of HRAS—MAPK-ERK due to the activation of stress response as suggested for paclitaxel [48] and cisplatin [49].

Following the induction of damage to cellular DNA, a major response is cell death due to apoptosis. Thus, induction of apoptosis as a possible outcome of DNA damage has been widely cited in the literature [50]. A morphological study with AO-EB double staining identified early, and late phases of apoptosis in isothiacalothrixin treated cells which also showed signs of nuclear condensation. Further, DNA fragmentation was demonstrated by agarose gel electrophoresis revealed that isothiacalothrixin B induced apoptosis in HCT116 cells. Furthermore, to study the ability of HCT116 cells to repair calothrixin induced DNA damage, a challenge assay was performed. The cells were treated with calothrixins for a short period to induce DNA damage and allowed to recover in drug-free media. The cell cycle analysis at different time points in the recovery period indicated that isothiacalothrixins maintained arrest of cells until 96 hours in G1 and G2/M phases while thiacalothrixin B or calothrixin B treated cells have normal cell cycle distribution. Similarly, comet assay at 48 hours during the recovery period too showed the appearance of comet tails in isothiacalothrixin treated cells but not with thiacalothrixin B treatment indicating that the former analogues caused irreparable DNA damage to HCT116 cells. The above result clearly suggests that the integrity of DNA is severely compromised immediately after treatment with **SCAB 4** which eventually leads to cell cycle arrest in S-G2/M phases which is maintained for a longer period and failure to repair damaged DNA, ultimately leads to apoptotic cell death in HCT116 cells.

## Conclusion

In conclusion, this study recommends that isothiacalothrixin B analogues be considered as a potent DNA damaging agent that caused apoptosis in HCT116 cell line. Given the fact that p53 is frequently mutated in tumours, isothiacalothrixin analogues whose cell killing was independent of p53 function could be a suitable clinical candidate for anti-cancer therapy. The isothiacalothrixin analogues inhibited the clonogenicity and caused strong arrest of cells in S, G2/M phases of cell cycle in HCT116 cell line. Further, these analogues readily cleaved plasmid DNA in the presence of a reductant and caused DNA strand breaks in HCT116 cells leading to cell death through apoptosis. Also, this study identified p21^waf1/cip1^ as a mediator of cellular sensitivity to thiacalothrixin B or calothrixin B. Apart from showing preference of DNA binding to AT-rich regions; the present study also provides the basis for irreparable DNA damage as a mechanism of isothiacalothrixin induced apoptosis making these analogues as promising anti-cancer agents.

## Supporting information

S1 Fig Isothiacalothrixins inhibited the cell viability of HCT116 cells over an extended period. Cell viability at various time points as assessed by Trypan blue staining of HCT-116 cells following treatment with different calothrixin analogues (5 μM) and camptothecin (0.5 μM). **SCAB 1** (solid black square), **SCAB 4** (solid red circle), **SCAB 6** (solid green triangle), **SCAB 10** (solid inverted magenta triangle), camptothecin (solid cyan diamond).
(TIF)

S2 Fig Clonogenic survival curves of HCT-116 and NCI-H460 cells. Attached cells were treated with 0.1–5 μM of calothrixin B, thiacalothrixin B and isothiacalothrixins (**SCAB 4**, **6** and **10**), for 48 hours. After 14 days, surviving colonies were fixed and stained with crystal violet. Error bars represent the standard error of the mean of three independent experiments.
(TIF)

S3 Fig DNA plasmid damage induced by different thiacalothrixin B analogues (SCAB 1–12). Experiments were performed in triplicate, and the results are expressed in the form of histograms representing the mean ± standard deviation of the percentage of the linear plasmid DNA form observed.
(TIF)

S4 Fig Calothrixin B and its isothia analogue caused little chromic shifts. Effects of increasing concentrations of CT-DNA on the UV-Vis absorption spectra of calothrixin B or thia calothrixin B analogues. Conditions: C_calothrixin B or thiacalothrixin B_, 3×10^−5^mol L^−1^; C_ctDNA_ (×10^−6^mol L^−1^); a→o: 0; 2; 5; 10; 15; 20; 25; 30; 35; 40; 45; 50; 60; 80; 100. The arrow shows the intensity changes in increasing CT-DNA concentration.
(TIF)

S5 Fig Isothiacalothrixin B (SCAB 4) caused irreparable DNA strand breaks in HCT116 cells. Single-cell gel electrophoresis data (comet assay) in HCT116 cells (A) treated for 4 hours with 5 μM thiacalothrixin B (**SCAB 1**), followed by growth in drug free medium for 48 hours (B), treated for 48 hours with 5 μM thiacalothrixin B (B), treated for 4 hours with 5 μM isothiacalothrixin B, (**SCAB 4**) followed by growth in drug free medium for 48 hours (D) and treated for 48 hours with 5 μM isothiacalothrixin B (E). The Olive tail moment (OTM) and mean percentage tail DNA (TDNA) in HCT116 cells exposed to 5 μM of SCAB1 or SCAB4 at above time points. Values are represented as Mean ± standard deviation. *, *P < 0*.*05*. Experiments were performed in duplicate.
(TIF)

S6 Fig Isothiacalothrixin B (SCAB 4) affected clonogenicity after 4 hours treatment to HCT-116 cells. Effects of thiacalothrixin B (**SCAB 1**) and isothiacalothrixin B (**SCAB 4**) on the clonogenic growth of colon adenocarcinoma HCT116. Cells were seeded in a six-well plate and after over-night adherence, treated with different concentrations (0.1–5 μM) of thiacalothrixin B and isothiacalothrixins B (**SCAB 4**), for 4 or 48 hours. After drug treatment, the cells were washed with Dulbecco’s phosphate buffered saline and let grow up to 14 days in drug-free medium. Cell colonies were stained with crystal violet and photographed.
(TIF)

S1 Table Structural details of thia analogues of calothrixin B with their code names. (DOCX)

## References

[1] Rickards RW, Rothschild JM, Willis AC, de CNM, Kirk J, Kirk K, et al Calothrixins A and B, novel pentacyclic metabolites from Calothrix cyanobacteria with potent activity against malaria parasites and human cancer cells. Tetrahedron. 1999;55:13513–20.

[2] Muthu Ramalingam B, Dhatchana Moorthy N, Chowdhury SR, Mageshwaran T, Vellaichamy E, Saha S, et al Synthesis and Biological Evaluation of Calothrixins B and their Deoxygenated Analogues. J Med Chem. 2018;61(3):1285–315. Epub 2018/01/10. 10.1021/acs.jmedchem.7b01797 . 29313676

[3] Beno BR, Yeung KS, Bartberger MD, Pennington LD, Meanwell NA. A Survey of the Role of Noncovalent Sulfur Interactions in Drug Design. J Med Chem. 2015;58(11):4383–438. Epub 2015/03/04. 10.1021/jm501853m . 25734370

[4] Politzer P, Murray JS. A Unified View of Halogen Bonding, Hydrogen Bonding and Other σ-Hole Interactions In: Scheiner S, editor. Noncovalent Forces. Cham: Springer International Publishing; 2015 p. 291–321.

[5] Fujiwara AN, Acton EM, Goodman L. N-Oxides and S-oxides of ellipticine analogs. J Het Chem. 1969;6(3):389–92. 10.1002/jhet.5570060318

[6] Muthu Ramalingam B, Dhatchana Moorthy N, Vellaichamy E, Mohanakrishnan AK. Synthesis of Thia-Analogues of Calothrixin B Involving FeCl3-Mediated Domino Reaction. Synlett. 2016;28(01):133–7. 10.1055/s-0036-1588072

[7] Skehan P, Storeng R, Scudiero D, Monks A, McMahon J, Vistica D, et al New colorimetric cytotoxicity assay for anticancer-drug screening. J Natl Cancer Inst. 1990;82(13):1107–12. Epub 1990/07/04. . 2359136 10.1093/jnci/82.13.1107

[8] Vichai V, Kirtikara K. Sulforhodamine B colorimetric assay for cytotoxicity screening. Nat Protoc. 2006;1(3):1112–6. Epub 2007/04/05. 10.1038/nprot.2006.179 . 17406391

[9] Franken NA, Rodermond HM, Stap J, Haveman J, van Bree C. Clonogenic assay of cells in vitro. Nat Protoc. 2006;1(5):2315–9. Epub 2007/04/05. 10.1038/nprot.2006.339 . 17406473

[10] Nandhakumar S, Parasuraman S, Shanmugam MM, Rao KR, Chand P, Bhat BV. Evaluation of DNA damage using single-cell gel electrophoresis (Comet Assay). J Pharmacol Pharmacother. 2011;2(2):107–11. Epub 2011/07/21. 10.4103/0976-500X.81903 . 21772771 PMC3127337

[11] Kumaravel TS, Vilhar B, Faux SP, Jha AN. Comet Assay measurements: a perspective. Cell Biol Toxicol. 2009;25(1):53–64. Epub 2007/11/28. 10.1007/s10565-007-9043-9 . 18040874

[12] Anantharaman A, Priya RR, Hemachandran H, Sivaramakrishna A, Babu S, Siva R. Studies on interaction of norbixin with DNA: multispectroscopic and in silico analysis. Spectrochim Acta A Mol Biomol Spectrosc. 2015;144:163–9. Epub 2015/03/11. 10.1016/j.saa.2015.02.049 . 25754392

[13] Ghosh P, Devi GP, Priya R, Amrita A, Sivaramakrishna A, Babu S, et al Spectroscopic and in silico evaluation of interaction of DNA with six anthraquinone derivatives. Appl Biochem Biotechnol. 2013;170(5):1127–37. Epub 2013/05/07. 10.1007/s12010-013-0259-2 . 23645388

[14] Bhakta D, Siva R. Morindone, an anthraquinone, intercalates DNA sans toxicity: a spectroscopic and molecular modeling perspective. Appl Biochem Biotechnol. 2012;167(4):885–96. Epub 2012/05/29. 10.1007/s12010-012-9744-2 . 22639367

[15] Lian C, Robinson H, Wang AHJ. Structure of Actinomycin D bound with (GAAGCTTC)2and (GATGCTTC)2and Its Binding to the (CAG)n:(CTG)nTriplet Sequence As Determined by NMR Analysis. J Am Chem Soc. 1996;118(37):8791–801. 10.1021/ja961631p

[16] Li Z, Wan H, Shi Y, Ouyang P. Personal experience with four kinds of chemical structure drawing software: review on ChemDraw, ChemWindow, ISIS/Draw, and ChemSketch. J Chem Inf Comput Sci. 2004;44(5):1886–90. Epub 2004/09/28. 10.1021/ci049794h . 15446849

[17] Pettersen EF, Goddard TD, Huang CC, Couch GS, Greenblatt DM, Meng EC, et al UCSF Chimera—a visualization system for exploratory research and analysis. J Comput Chem. 2004;25(13):1605–12. Epub 2004/07/21. 10.1002/jcc.20084 . 15264254

[18] Morris GM, Huey R, Lindstrom W, Sanner MF, Belew RK, Goodsell DS, et al AutoDock4 and AutoDockTools4: Automated docking with selective receptor flexibility. J Comput Chem. 2009;30(16):2785–91. Epub 2009/04/29. 10.1002/jcc.21256 . 19399780 PMC2760638

[19] Lill MA, Danielson ML. Computer-aided drug design platform using PyMOL. J Comput Aided Mol Des. 2011;25(1):13–9. Epub 2010/11/06. 10.1007/s10822-010-9395-8 . 21053052

[20] Sastry GM, Adzhigirey M, Day T, Annabhimoju R, Sherman W. Protein and ligand preparation: parameters, protocols, and influence on virtual screening enrichments. J Comput Aided Mol Des. 2013;27(3):221–34. Epub 2013/04/13. 10.1007/s10822-013-9644-8 . 23579614

[21] Neidle S, Mann J, Rayner EL, Baron A, Opoku-boahen Y, Simpson IJ, et al Symmetric bis-benzimidazoles: new sequence-selective DNA-binding molecules. Chem Comm. 1999;(10):929–30. 10.1039/a901074b

[22] Friesner RA, Banks JL, Murphy RB, Halgren TA, Klicic JJ, Mainz DT, et al Glide: a new approach for rapid, accurate docking and scoring. 1. Method and assessment of docking accuracy. J Med Chem. 2004;47(7):1739–49. Epub 2004/03/19. 10.1021/jm0306430 . 15027865

[23] Halgren TA, Murphy RB, Friesner RA, Beard HS, Frye LL, Pollard WT, et al Glide: a new approach for rapid, accurate docking and scoring. 2. Enrichment factors in database screening. J Med Chem. 2004;47(7):1750–9. Epub 2004/03/19. 10.1021/jm030644s . 15027866

[24] Kasibhatla S, Amarante-Mendes GP, Finucane D, Brunner T, Bossy-Wetzel E, Green DR. Acridine Orange/Ethidium Bromide (AO/EB) Staining to Detect Apoptosis. CSH Protoc. 2006;2006(3):pdb.prot4493. Epub 2006/01/01. 10.1101/pdb.prot4493 . 22485874

[25] Herrmann M, Lorenz HM, Voll R, Grunke M, Woith W, Kalden JR. A rapid and simple method for the isolation of apoptotic DNA fragments. Nucleic Acids Res. 1994;22(24):5506–7. Epub 1994/12/11. . 7816645 10.1093/nar/22.24.5506 PMC332111

[26] el-Deiry WS, Tokino T, Velculescu VE, Levy DB, Parsons R, Trent JM, et al WAF1, a potential mediator of p53 tumor suppression. Cell. 1993;75(4):817–25. Epub 1993/11/19. 10.1016/0092-8674(93)90500-p . 8242752

[27] Azqueta A, Shaposhnikov S, C AR. DNA Repair Measured by the Comet Assay. DNA Repair: InTech; 2011 p. 615–36.

[28] Pett L, Hartley JA, Kiakos K. Therapeutic Agents Based on DNA Sequence Specific Binding. Curr Top Med Chem. 2015;15(14):1293–322. Epub 2015/04/14. . 25866278 10.2174/1568026615666150413155431

[29] Haq I, Ladbury J. Drug-DNA recognition: energetics and implications for design. J Mol Recognit. 2000;13(4):188–97. Epub 2000/08/10. 10.1002/1099-1352(200007/08)13:4<188::AID-JMR503>3.0.CO;2-1 . 10931556

[30] Dickerson R, Drew H, Conner B, Wing R, Fratini A, Kopka M. The anatomy of A-, B-, and Z-DNA. Science. 1982;216(4545):475–85. 10.1126/science.7071593 7071593

[31] Dickerson RE. DNA structure from A to Z Methods in Enzymology. 211: Academic Press; 1992 p. 67–111. 1406328 10.1016/0076-6879(92)11007-6

[32] Ricci CG, Netz PA. Docking studies on DNA-ligand interactions: building and application of a protocol to identify the binding mode. J Chem Inf Model. 2009;49(8):1925–35. Epub 2009/08/07. 10.1021/ci9001537 . 19655805

[33] Hannon MJ. Supramolecular DNA recognition. Chem Soc Rev. 2007;36(2):280–95. Epub 2007/02/01. 10.1039/b606046n . 17264930

[34] Sugawara E, Nikaido H. Properties of AdeABC and AdeIJK efflux systems of Acinetobacter baumannii compared with those of the AcrAB-TolC system of Escherichia coli. Antimicrob Agents Chemother. 2014;58(12):7250–7. Epub 2014/09/24. 10.1128/AAC.03728-14 . 25246403 PMC4249520

[35] Miller CM, O’Sullivan EC, Devine KJ, McCarthy FO. Synthesis and biological evaluation of novel isoellipticine derivatives and salts. Org Biomol Chem. 2012;10(39):7912–21. Epub 2012/09/04. 10.1039/c2ob26181b . 22940706

[36] Russell EG, O’Sullivan EC, Miller CM, Stanicka J, McCarthy FO, Cotter TG. Ellipticine derivative induces potent cytostatic effect in acute myeloid leukaemia cells. Invest New Drugs. 2014;32(6):1113–22. Epub 2014/08/12. 10.1007/s10637-014-0140-3 . 25107543

[37] Russell EG, Guo J, O’Sullivan EC, O’Driscoll CM, McCarthy FO, Cotter TG. 7-formyl-10-methylisoellipticine, a novel ellipticine derivative, induces mitochondrial reactive oxygen species (ROS) and shows anti-leukaemic activity in mice. Invest New Drugs. 2016;34(1):15–23. Epub 2015/11/13. 10.1007/s10637-015-0302-y . 26559431

[38] Sancar A, Lindsey-Boltz LA, Unsal-Kacmaz K, Linn S. Molecular mechanisms of mammalian DNA repair and the DNA damage checkpoints. Annu Rev Biochem. 2004;73(0066–4154 (Print)):39–85. Epub 2004/06/11. 10.1146/annurev.biochem.73.011303.073723 . 15189136

[39] Tian K, Rajendran R, Doddananjaiah M, Krstic-Demonacos M, Schwartz JM. Dynamics of DNA damage induced pathways to cancer. PLoS One. 2013;8(9):e72303 Epub 2013/09/12. 10.1371/journal.pone.0072303 . 24023735 PMC3762865

[40] Olive PL, Banath JP. The comet assay: a method to measure DNA damage in individual cells. Nat Protoc. 2006;1(1):23–9. Epub 2007/04/05. 10.1038/nprot.2006.5 . 17406208

[41] Bischoff G, Hoffmann S. DNA-binding of drugs used in medicinal therapies. Curr Med Chem. 2002;9(3):312–48. 11860361 10.2174/0929867023371085

[42] Lane DP. Cancer. p53, guardian of the genome. Nature. 1992;358(6381):15–6. Epub 1992/07/02. 10.1038/358015a0 . 1614522

[43] Melino G, Bernassola F, Ranalli M, Yee K, Zong WX, Corazzari M, et al p73 Induces apoptosis via PUMA transactivation and Bax mitochondrial translocation. J Biol Chem. 2004;279(9):8076–83. Epub 2003/11/25. 10.1074/jbc.M307469200 . 14634023

[44] Mrozek-Wilczkiewicz A, Spaczynska E, Malarz K, Cieslik W, Rams-Baron M, Krystof V, et al Design, Synthesis and In Vitro Activity of Anticancer Styrylquinolines. The p53 Independent Mechanism of Action. PLoS One. 2015;10(11):e0142678 Epub 2015/11/26. 10.1371/journal.pone.0142678 . 26599982 PMC4657899

[45] Bauer MK, Vogt M, Los M, Siegel J, Wesselborg S, Schulze-Osthoff K. Role of reactive oxygen intermediates in activation-induced CD95 (APO-1/Fas) ligand expression. J Biol Chem. 1998;273(14):8048–55. Epub 1998/05/09. 10.1074/jbc.273.14.8048 . 9525905

[46] Ho JQ, Asagiri M, Hoffmann A, Ghosh G. NF-kappaB potentiates caspase independent hydrogen peroxide induced cell death. PLoS One. 2011;6(2):e16815 Epub 2011/02/25. 10.1371/journal.pone.0016815 . 21347231 PMC3039651

[47] Breitschopf K, Haendeler J, Malchow P, Zeiher AM, Dimmeler S. Posttranslational modification of Bcl-2 facilitates its proteasome-dependent degradation: molecular characterization of the involved signaling pathway. Mol Cell Biol. 2000;20(5):1886–96. Epub 2000/02/12. 10.1128/mcb.20.5.1886-1896.2000 . 10669763 PMC85374

[48] Bacus SS, Gudkov AV, Lowe M, Lyass L, Yung Y, Komarov AP, et al Taxol-induced apoptosis depends on MAP kinase pathways (ERK and p38) and is independent of p53. Oncogene. 2001;20(2):147–55. Epub 2001/04/21. 10.1038/sj.onc.1204062 . 11313944

[49] Wang X, Martindale JL, Holbrook NJ. Requirement for ERK activation in cisplatin-induced apoptosis. J Biol Chem. 2000;275(50):39435–43. Epub 2000/09/20. 10.1074/jbc.M004583200 . 10993883

[50] Norbury CJ, Zhivotovsky B. DNA damage-induced apoptosis. Oncogene. 2004;23(16):2797–808. Epub 2004/04/13. 10.1038/sj.onc.1207532 . 15077143

