# Supplementary figures and images for: Novel isothiacalothrixin B analogues exhibit cytotoxic activity on human colon cancer cells in vitro by inducing irreversible DNA damage
Source: PLoS One. 2018 Sep 6;13(9):e0202903. doi: 10.1371/journal.pone.0202903 (PMC6126808; doi:10.1371/journal.pone.0202903)

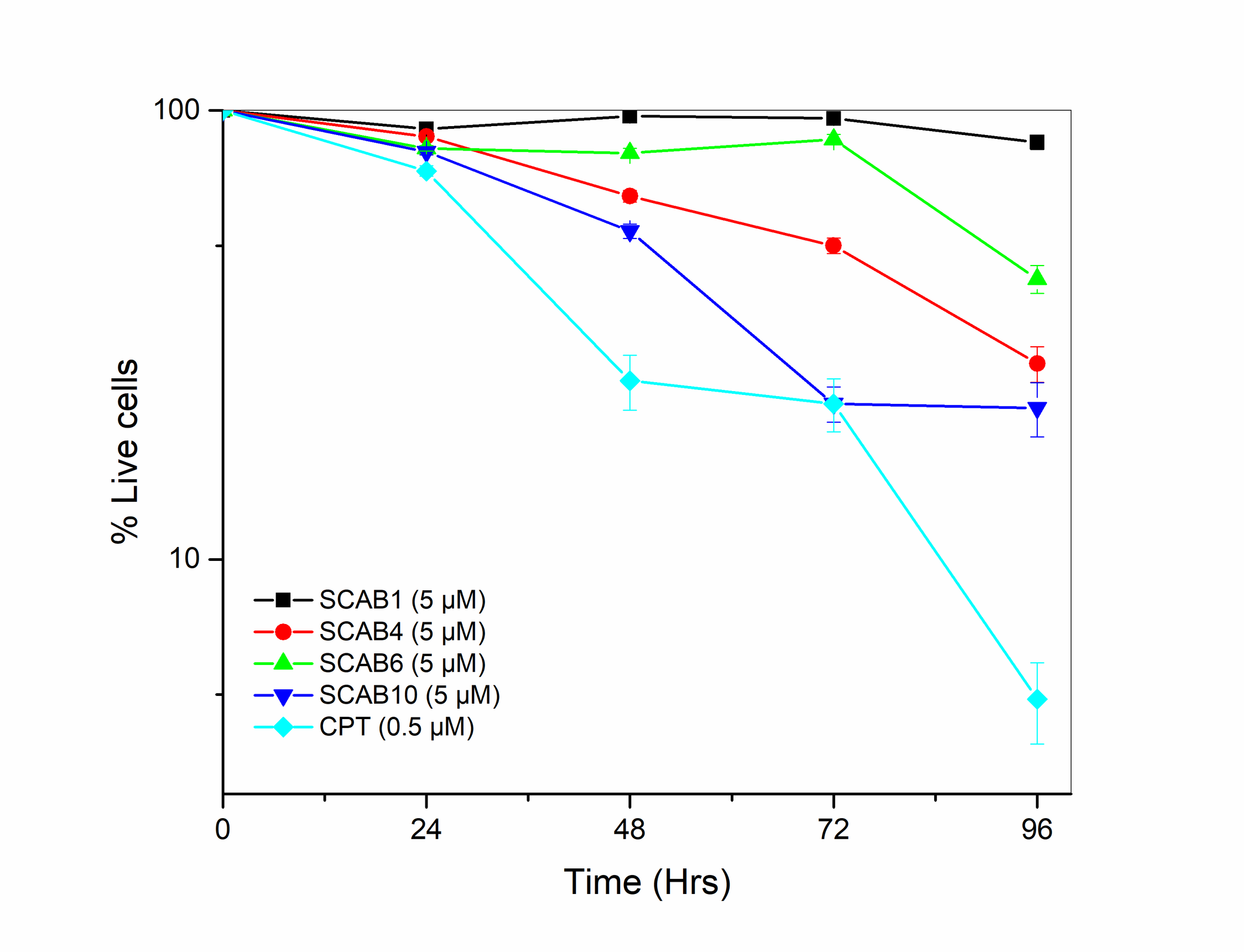

Supplement: S1 Fig — Cell viability at various time points as assessed by Trypan blue staining of HCT-116 cells following treatment with different calothrixin analogues (5 μM) and camptothecin (0.5 μM). SCAB 1 (solid black square), SCAB 4 (solid red circle), SCAB 6 (solid green triangle), SCAB 10 (solid inverted magenta triangle), camptothecin (solid cyan diamond). (TIF) [file pone.0202903.s001.tif]

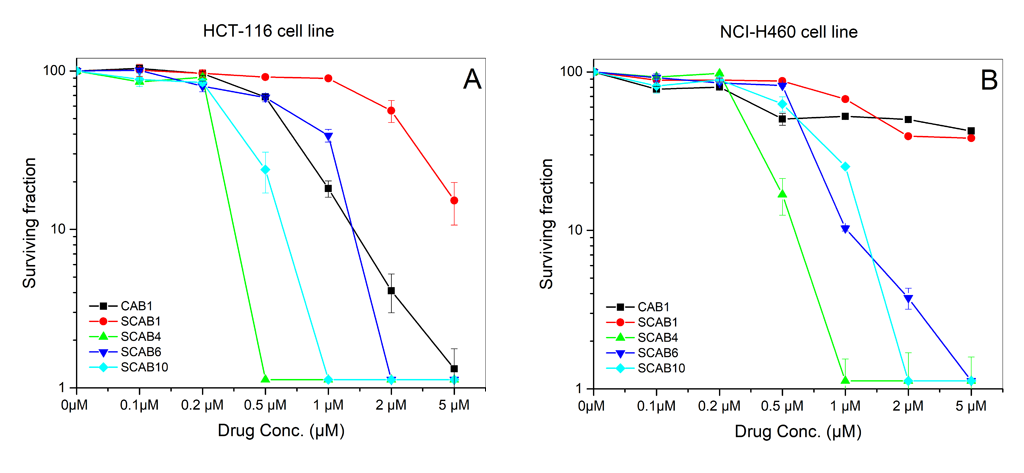

Supplement: S2 Fig — Attached cells were treated with 0.1–5 μM of calothrixin B, thiacalothrixin B and isothiacalothrixins (SCAB 4, 6 and 10), for 48 hours. After 14 days, surviving colonies were fixed and stained with crystal violet. Error bars represent the standard error of the mean of three independent experiments. (TIF) [file pone.0202903.s002.tif]

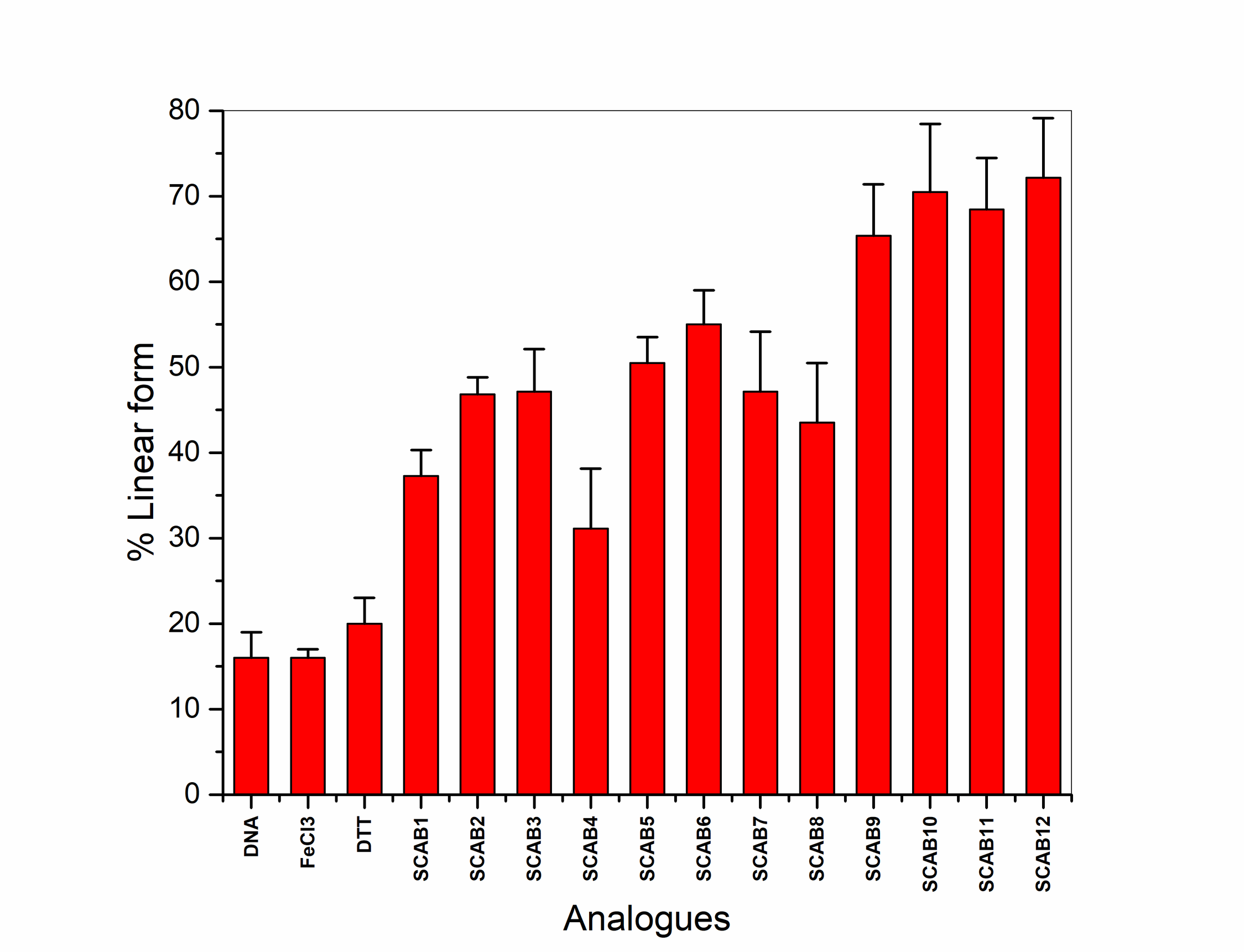

Supplement: S3 Fig — Experiments were performed in triplicate, and the results are expressed in the form of histograms representing the mean ± standard deviation of the percentage of the linear plasmid DNA form observed. (TIF) [file pone.0202903.s003.tif]

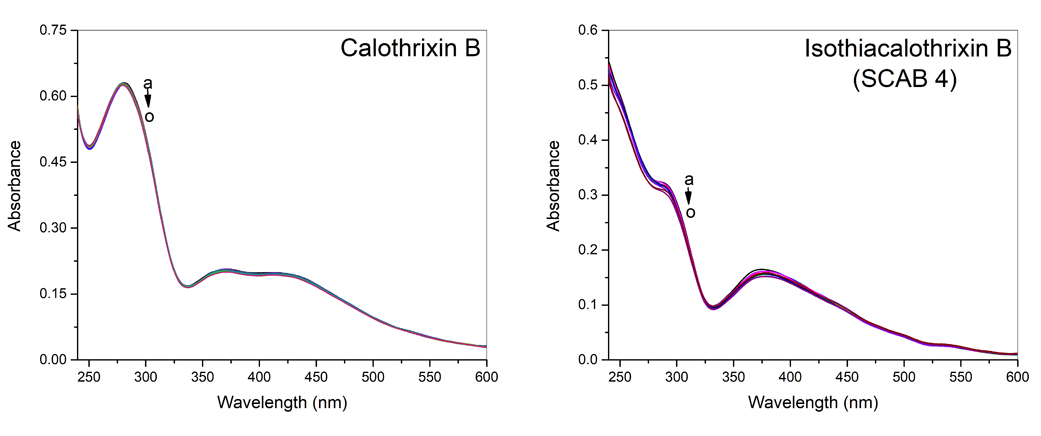

Supplement: S4 Fig — Effects of increasing concentrations of CT-DNA on the UV-Vis absorption spectra of calothrixin B or thia calothrixin B analogues. Conditions: Ccalothrixin B or thiacalothrixin B, 3×10^−5^mol L−1; CctDNA (×10−6mol L−1); a→o: 0; 2; 5; 10; 15; 20; 25; 30; 35; 40; 45; 50; 60; 80; 100. The arrow shows the intensity changes in increasing CT-DNA concentration. (TIF) [file pone.0202903.s004.tif]

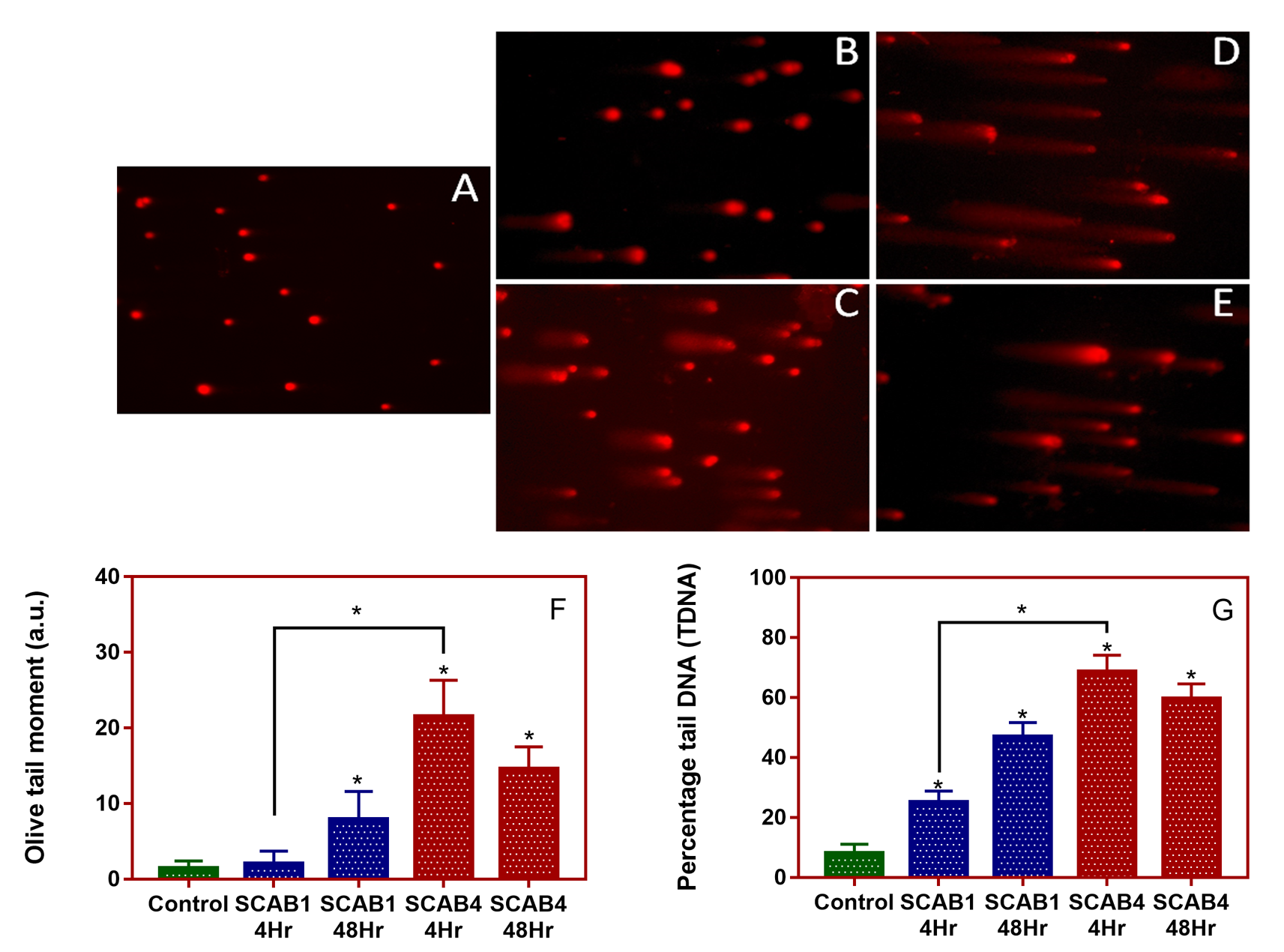

Supplement: S5 Fig — Single-cell gel electrophoresis data (comet assay) in HCT116 cells (A) treated for 4 hours with 5 μM thiacalothrixin B (SCAB 1), followed by growth in drug free medium for 48 hours (B), treated for 48 hours with 5 μM thiacalothrixin B (B), treated for 4 hours with 5 μM isothiacalothrixin B, (SCAB 4) followed by growth in drug free medium for 48 hours (D) and treated for 48 hours with 5 μM isothiacalothrixin B (E). The Olive tail moment (OTM) and mean percentage tail DNA (TDNA) in HCT116 cells exposed to 5 μM of SCAB1 or SCAB4 at above time points. Values are represented as Mean ± standard deviation. *, P < 0.05. Experiments were performed in duplicate. (TIF) [file pone.0202903.s005.tif]

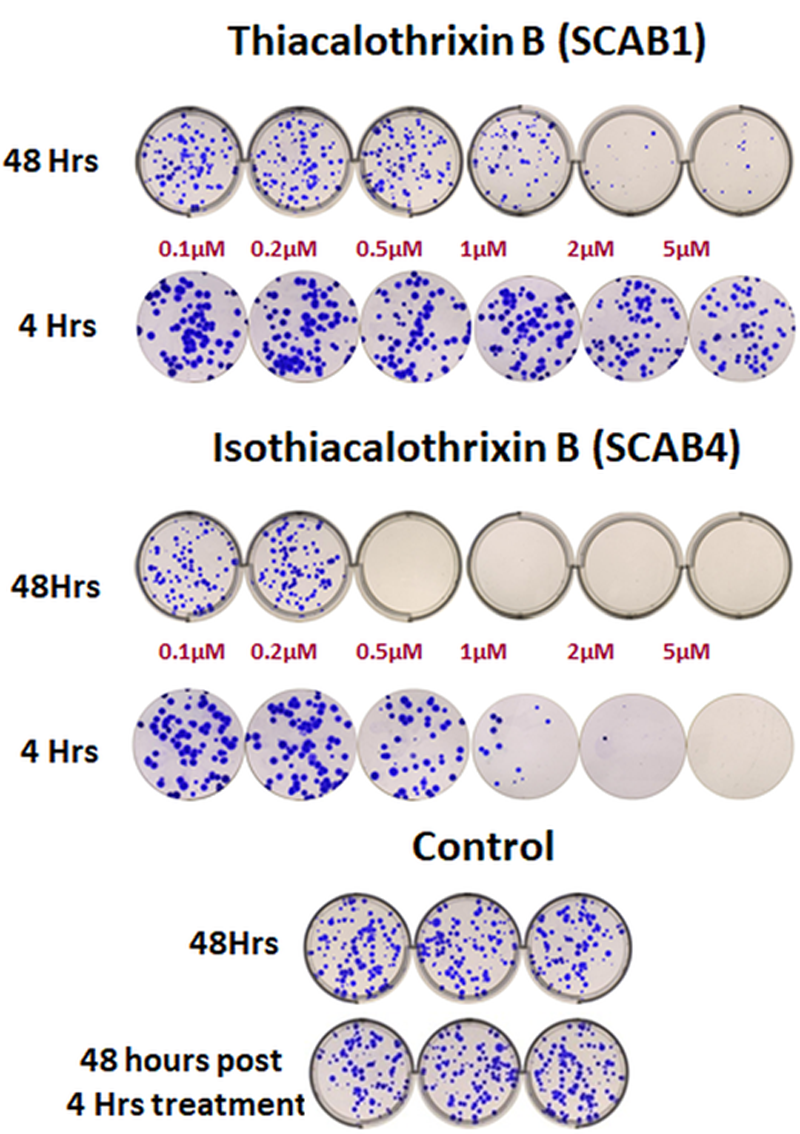

Supplement: S6 Fig — Effects of thiacalothrixin B (SCAB 1) and isothiacalothrixin B (SCAB 4) on the clonogenic growth of colon adenocarcinoma HCT116. Cells were seeded in a six-well plate and after over-night adherence, treated with different concentrations (0.1–5 μM) of thiacalothrixin B and isothiacalothrixins B (SCAB 4), for 4 or 48 hours. After drug treatment, the cells were washed with Dulbecco’s phosphate buffered saline and let grow up to 14 days in drug-free medium. Cell colonies were stained with crystal violet and photographed. (TIF) [file pone.0202903.s006.tif]
