# Supplementary material for: Novel isothiacalothrixin B analogues exhibit cytotoxic activity on human colon cancer cells in vitro by inducing irreversible DNA damage
Source: PLoS One. 2018 Sep 6;13(9):e0202903. doi: 10.1371/journal.pone.0202903 (PMC6126808; doi:10.1371/journal.pone.0202903)
Supplement: S1 Table — (DOCX) [file pone.0202903.s007.docx]

|  | | | |
| --- | --- | --- | --- |
| Sl. No | Name of the thiacalothrixin B analogues | R_1_ | |
| 1 | SCAB1 | H | |
| 2 | SCAB2 | Cl | |
| 3 | SCAB3 | F | |
|  | | | |
| Sl. No. | Name of the isothiacalothrixin B analogues | R_1_ | R_2_ |
| 4 | SCAB4 | H | H |
| 5 | SCAB5 | H | Cl |
| 6 | SCAB6 | H | F |
| 7 | SCAB7 | Cl | H |
| 8 | SCAB8 | Cl | Cl |
| 9 | SCAB9 | Cl | F |
| 10 | SCAB10 | F | H |
| 11 | SCAB11 | F | Cl |
| 12 | SCAB12 | F | F |

**S1 Table.** Structural details of thia analogues of calothrixin B with their code names
